# Binding free energies in the SAMPL5 octa-acid host–guest challenge calculated with DFT-D3 and CCSD(T)

**DOI:** 10.1007/s10822-016-9957-5

**Published:** 2016-09-06

**Authors:** Octav Caldararu, Martin A. Olsson, Christoph Riplinger, Frank Neese, Ulf Ryde

**Affiliations:** 1 Department of Theoretical Chemistry, Chemical Centre, Lund University, P. O. Box 124, 221 00 Lund, Sweden; 2 Max-Planck-Institut für Chemische Energiekonversion, Stiftstraße 34-36, 45470 Mülheim an der Ruhr, Germany

**Keywords:** Ligand-binding affinities, Host–guest systems, Density-functional theory, Dispersion corrections, COSMO-RS, DLPNO–CCSD(T), SAMPL5

## Abstract

We have tried to calculate the free energy for the binding of six small ligands to two variants of the octa-acid deep cavitand host in the SAMPL5 blind challenge. We employed structures minimised with dispersion-corrected density-functional theory with small basis sets and energies were calculated using large basis sets. Solvation energies were calculated with continuum methods and thermostatistical corrections were obtained from frequencies calculated at the HF-3c level. Care was taken to minimise the effects of the flexibility of the host by keeping the complexes as symmetric and similar as possible. In some calculations, the large net charge of the host was reduced by removing the propionate and benzoate groups. In addition, the effect of a restricted molecular dynamics sampling of structures was tested. Finally, we tried to improve the energies by using the DLPNO–CCSD(T) approach. Unfortunately, results of quite poor quality were obtained, with no correlation to the experimental data, systematically too positive affinities (by ~50 kJ/mol) and a mean absolute error (after removal of the systematic error) of 11–16 kJ/mol. DLPNO–CCSD(T) did not improve the results, so the accuracy is not limited by the energy function. Instead, four likely sources of errors were identified: first, the minimised structures were often incorrect, owing to the omission of explicit solvent. They could be partly improved by performing the minimisations in a continuum solvent with four water molecules around the charged groups of the ligands. Second, some ligands could bind in several different conformations, requiring sampling of reasonable structures. Third, there is an indication the continuum-solvation model has problems to accurately describe the binding of both the negatively and positively charged guest molecules. Fourth, different methods to calculate the thermostatistical corrections gave results that differed by up to 30 kJ/mol and there is an indication that HF-3c overestimates the entropy term. In conclusion, it is a challenge to calculate binding affinities for this octa-acid system with quantum–mechanical methods.

**Electronic supplementary material:**

The online version of this article (doi:10.1007/s10822-016-9957-5) contains supplementary material, which is available to authorized users.

## Introduction

One of the most important challenges for computational chemistry is to accurately predict the free energy for the binding of a small molecule to a biomacromolecule. This could involve the binding of a drug candidate to its target receptor, having obvious applications in pharmaceutical chemistry. Consequently, a large number of methods have been developed for this purpose, including statistics-based docking and scoring methods, molecular-mechanics (MM) simulations, and free-energy simulations (FES) [1–6]. The binding free energy has contributions from a large number of interactions, such as bonded terms, dispersion, exchange-repulsion, electrostatics, polarisation, charge transfer, charge penetration, solvation, and entropy. As MM force fields have inherent limitations in treating several of these interactions, there has been a growing interest in using quantum–mechanical (QM) methods to improve binding-affinity calculations [7–14].

Protein–ligand complexes are very large, involving thousands of atoms and often present major problems in predicting binding affinities, e.g. owing to conformational changes of the protein during ligand binding or changes in the protonation states of the ligand and the receptor. In contrast, organic macrocycles with a few hundred atoms has a much smaller configurational freedom and chemical diversity. Still, the binding of small molecules to such systems involve the same type of interactions as protein–ligand binding, allowing the study of ligand binding in a simpler context. Therefore, there has been quite some interest in such host–guest systems in recent years [15–20].

In particular, host–guest systems have been studied in blind-test challenges, in which the experimental binding affinity are not known beforehand, which reduces bias against the experimental data. For example, the SAMPL3 blind test involved the binding of eleven different guest molecules to three host molecules [21]. Ten research groups provided predictions but none of them could obtain both a good correlation and a low root-mean-squared deviation (RMSD) from the experimental binding affinities.

In the SAMPL4 competition, two hosts were involved, together with 25 guest ligands [22]. For the curcurbit [7] uril host, the best results were obtained with either FES or the much simpler and faster solvent interaction-energy method [23], both at the MM level of theory, with RMSD of 8 kJ/mol and a correlation coefficient (*R* ^2^) of 0.6–0.8 [22, 24]. For the octa-acid deep-cavity host [25, 26], even better results were obtained by FES calculations at the MM level, giving a correlation (*R* ^2^) of 0.9 and RMSD of 4 kJ/mol [22, 27]. This was partly owing to the fact that the ligands were ideally suited for FES calculations of relative energies, with a high degree of similarity and a conserved single negative charge.

**Fig. 1:**
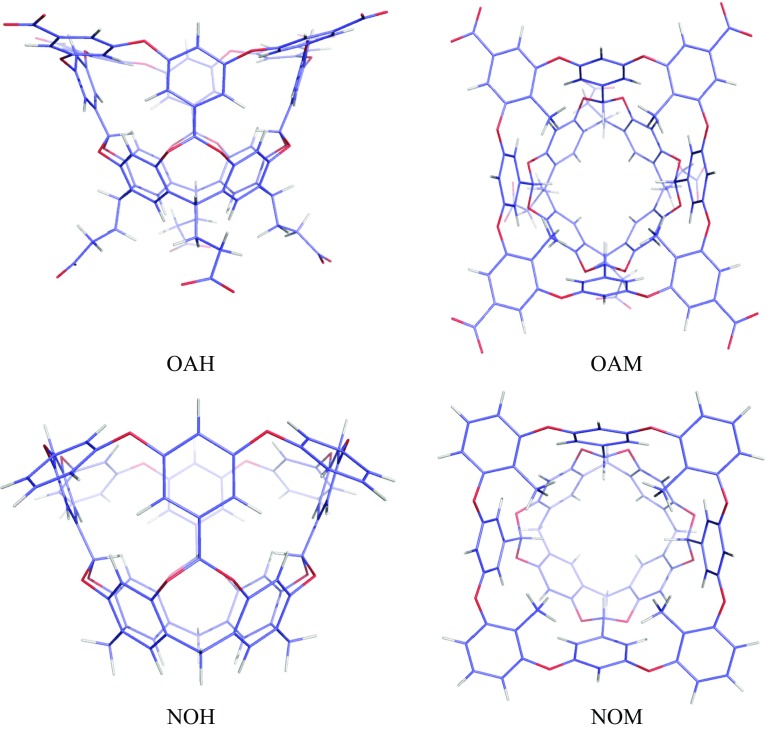
The octa-acid host (OAH, *side view*) and the methylated octa-acid host (OAM, *top view*), as well as the corresponding truncated hosts, NOH and NOM

**Fig. 2:**
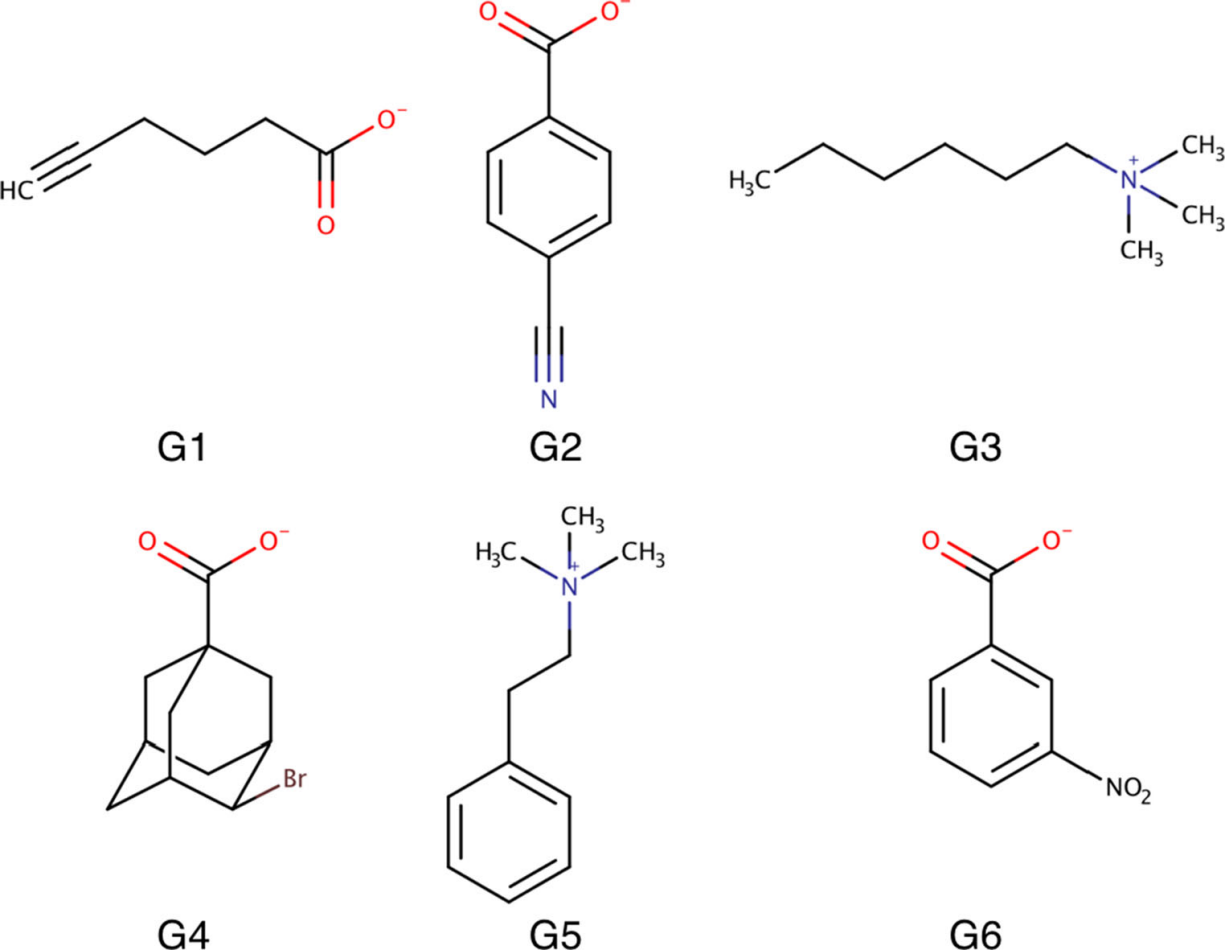
The six guest molecules, *G1*–*G6*

In this paper we present our attempts to predict the binding affinity for two variants of the octa-acid host–guest system [28] included in the SAMPL5 challenge (Fig. 1) [29]. The six ligands involved in the challenge (shown in Fig. 2) are quite dissimilar and have a varying net charge (−1 or +1), and are therefore less suited for the FES method for relative free energies successfully used in the SAMPL4 challenge [22, 27]. Instead, we decided to try to improve the QM method originally suggested by Grimme [30, 31], which employs structures optimised by dispersion-corrected density-functional theory (DFT-D) [32], as well as continuum solvation and entropies from normal-mode calculations. Both we and Grimme used such an approach for octa-acid challenge in SAMPL4 with intermediate results (*R* ^2^ = 0.6–0.8 and a mean absolute deviation, MAD, of 5–9 kJ/mol) [27, 33]. For the curcurbit [7] uril host in SAMPL4, the method gave among the best results with *R* ^2^ = 0.8 and RMSD = 10 kJ/mol. Grimme and coworkers have applied this approach for 30 host–guest systems, giving a MAD of 10 kJ/mol for absolute binding free energies and only 5 kJ/mol for relative energies of pairs of guest molecules binding to the same host [13].

We have tried to improve the approach in three aspects: by controlling the structural variation, by reducing the charge and flexibility of the ligand, and by employing a restricted molecular dynamics sampling. In addition, we also tested to improve the QM calculations with the domain-based local pair natural orbital coupled-cluster singles and doubles with perturbatively treated triples approach (DLPNO–CCSD(T)) [34].

## Methods

### Studied systems

We have studied the two octa-acid host–guest systems [26, 35] in the SAMPL5 blind challenge [29], shown in Fig. 1. As the name indicates, the hosts have eight negative charges, four benzoic groups at the upper rim of the cavitand and four propionate groups at the lower part of the host. The chemical structure has a four-fold symmetry. The two hosts differ only in that the benzoic groups have either a hydrogen atom or a methyl group in the para position of the carboxylate group (i.e. the position directed towards the cavity). The two hosts will be abbreviated OAH and OAM in the following. Another set of hosts were constructed by replacing the benzoic carboxylate groups and the full propionate groups with hydrogen atoms. These neutralised hosts will be called NOH and NOM, depending on whether they carry the methyl groups or not. They are also shown in Fig. 1.

The six guest molecules are shown in Fig. 2 and will be called G1–G6 below. G1, G2, G4, and G6 have a carboxylic group and therefore a single negative charge. G2 and G6 have a benzoic group, like the nine guests in the SAMPL4 challenge. G1 instead has a hexyne group and G4 an adamantane group. The other two guest molecules, G3 and G5, have a trimethylammonium group, giving them a single positive charge (independent of pH). G3 contains a hexane chain, whereas G5 involves an ethylbenzene group. The binding affinities were measured at pH 11.3–11.5 in order to ensure that all carboxylic groups are fully charged [29].

Structures of the hosts, guests, and complexes were built manually, based on structures obtained by MM and QM for the SAMPL4 ligands. The isolated hosts were forced to be symmetric and for the complexes we also tried to keep an approximate symmetry, thereby making the structures as similar as possible.

### DFT-D3 calculations

All DFT calculation were performed with the TURBOMOLE 7.0 software [36, 37]. All structures (complexes, as well as isolated hosts and guests) were optimised with the TPSS-D3 method [38] and the def2-SV(P) basis set [39] in a vacuum. Dispersion was included by the DFT-D3 approach [40], with default damping. For each optimised structure, more accurate QM energies were calculated with both the TPSS and PBE [41] functionals and the def2-QZVP’ basis set, i.e. the def2-QZVP basis set [39] with the *f*-type functions on hydrogen and the *g*-type functions on the other atoms deleted [30]. In these calculations, DFT-D3 dispersion with Becke–Johnson damping and third-order terms included were calculated with the dftd3 software [42]. All DFT calculations were sped up by expanding the Coulomb interactions in auxiliary basis sets with the resolution-of-identity approximation (RI), using the corresponding auxiliary basis sets [43, 44]. The def2-QZVP’ calculations also employed the multipole-accelerated resolution-of-identity J approach [45].

Solvation free energies in water solution were calculated with the conductor-like solvent model (COSMO) [46, 47] real-solvent (COSMO-RS) approach [48, 49] using the COSMOTHERM software [50]. These calculations were based on two single-point BP86 [51, 52] calculations with the TZVP basis set [53], one performed in a vacuum and the other in the COSMO solvent with an infinite dielectric constant. For the OAH and OAM hosts with their extensive negative charge, we had to use the undocumented ADEG option to force the program to accept that the solvation energy is very large.

Thermal corrections to the Gibbs free energy (including the zero point vibrational energy) were calculated at 298 K and 1 atm pressure using an ideal-gas rigid-rotor harmonic-oscillator approach [54] from vibrational frequencies calculated at the HF-3c level [55] after a geometry optimisation at the same level of theory. The frequencies were scaled by a factor of 0.86 [30]. To obtain more stable results, low-lying vibrational modes (below 100 cm^−1^) were treated by a free-rotor approximation, using the interpolation model suggested by Grimme and implemented in the thermo program [30]. The translational entropy and therefore also the free energy were corrected by 7.9 kJ/mol for the change in the standard state from 1 atm (used in the thermo program) to 1 M (used in the experiments). For the symmetry number, it was assumed that all isolated hosts have a fourfold symmetry and that the isolated G2 has a twofold symmetry, whereas all other guests and the complexes have a unit symmetry number.

The final free energy was calculated as follows:

1 $$\Delta G_{\text{tot}} =\Delta E_{\text{QM}} +\Delta E_{\text{disp}} +\Delta G_{\text{solv}} +\Delta G_{\text{therm}}$$

where ∆*E*_QM_ is the TPSS/def2-QZVP’ energy, ∆*E*_disp_ is the dispersion energy, with Becke–Johnson damping, including third-order terms and parameters for the TPSS functional, Δ*G*_solv_ is the COSMO-RS solvation free energy, and ∆*G*_therm_ is the thermostatistical correction described above. The final binding affinity is the difference in this free energy between the complex, host, and guest:

2 $$\Delta G_{\text{bind}} =\Delta G_{\text{tot}} ({\text{complex}}) -\Delta G_{\text{tot}} ({\text{host}}) -\Delta G_{\text{tot}} ({\text{guest}})$$

Strictly, the binding free energy should be calculated for optimised structures of all three terms in this equation. However, more stable energies are obtained if the host and guest structures are taken from that in the complex by simply deleting the other moiety (rigid binding free energies) [27, 56]. The latter energies can be corrected by the guest relaxation energy (∆*E*_Grlx_), calculated at the TPSS/def2-QZVP’ level of theory.

### Coupled-cluster calculations

DLPNO–CCSD(T) calculations [57–59] were performed with a development version of the ORCA suite of programs (based on version 3.0.3) [60]. We used the def2-TZVPP and def2-QZVPP basis sets with the corresponding auxiliary basis sets [39, 53, 61]. For all calculations that involved ligand 4 (which contains bromide), the scalar-relativistic zeroth-order regular approximation (ZORA) [62] and consistently segmented all-electron relativistically contracted (SARC) basis sets were used [63]. Basis sets of atoms that belong to negatively charged functional groups were replaced by the corresponding minimally augmented basis sets [64]. All calculations were counterpoise corrected [65]. Hartree–Fock and correlation energies were extrapolated to the complete basis set limit [66]. A combination of NormalPNO thresholds (intramolecular interactions of host and guest molecule) and TightPNO thresholds (intermolecular interactions between host and guest molecule) were used [67, 68]. To obtain binding free energies, we simply replaced the ∆*E*_QM_ + ∆*E*_disp_ terms in Eq. 1 with the DLPNO–CCSD(T) energy.

### MD simulations

To study the structure of the complexes in water solution and to sample a set of relevant structures all twelve host–guest systems were studied by molecular dynamics (MD) simulations. The simulations were performed only for the NOH and NOM systems and they were started from the optimised TPSS-D3/def2-SV(P) structures. The hosts and guests were solvated in a truncated octahedral box of explicit TIP4P-Ewald water molecules [69] extending 10 Å from the solute using the tleap module, giving a total of 1120–1296 atoms.

All MD simulations were performed using the Amber 14 software [70] with the GAFF force field [71] for the host and ligands. Parameters for NOH have been described before [72] and the parameters for the other host and the guest molecules were determined in the same way: The molecules were geometry optimised at the AM1 [73] level, followed by a calculation of the electrostatic potential at the HF/6-31G* [74] level of theory at points sampled around the molecule according to the Merz–Kollman scheme [75]. These calculations were performed with the Gaussian09 [76] software. Finally, restrained electrostatic-potential (RESP) charges [77] were fitted to the electrostatic potential using the antechamber program in the Amber 14 suite. The charges were symmetrised to reflect the (approximate) *C*_4v_ symmetry of the host molecules. One missing dihedral parameter for G2 was obtained from vibrational frequencies calculated at the B3LYP/def2-SV(P) level of theory using the Seminario approach [78], implemented in the Hess2FF program [79]. The Amber topology files for NOM and the ligands, as well as the added force-field parameters are given in the Supplementary material.

In all simulations, periodic boundary conditions were employed. For each complex, 10,000 steps of minimisation were used, followed by 20 ps constant-volume equilibration and 2 ns constant-pressure equilibration. In order to allow for a time step of 2 ps, the SHAKE algorithm [80] was used to constrain bonds involving hydrogen atoms to their equilibrium values. The temperature was kept constant at 300 K using Langevin dynamics [81], with a collision frequency of 2 ps^−1^ and the pressure was kept constant at 1 atm using a weak-coupling isotropic algorithm [82] with a relaxation time of 1 ps. Long-range electrostatics were handled by the particle-mesh Ewald (PME) method [83] with a fourth-order B spline interpolation and a tolerance of 10^−5^. The cut-off radius for Lennard–Jones interactions was set to 8 Å. No counter-ions were used in the calculations, because we have previously shown that they only have a minor (~2 kJ/mol) influence on the binding free energies [84].

In the first simulations, G4 dissociated from the OAM host. Therefore, a restraint of 209 kJ/mol/Å^2^ was added between one of the hydrogen atoms of the host that points into the cavity and the Br atom of the guest. This ensured that the guest stayed inside the host throughout the simulation.

Previous FES calculations for the nine SAMPL4 ligands of the OAH host have shown that the deletion of the benzoic and propionate groups have only minor influence on the relative binding free energies (less than 2 kJ/mol difference for the relative free energies) [72]. We tested also an intermediate host molecule, still with benzoic groups, but with the propionate groups removed. However, it gave almost identical results to the NOH host (within 1 kJ/mol; shown in Table S1 in the Supplementary material). Therefore, this host molecule was not further tested for the SAMPL5 ligands.

### Geometric measures

**Fig. 3:**
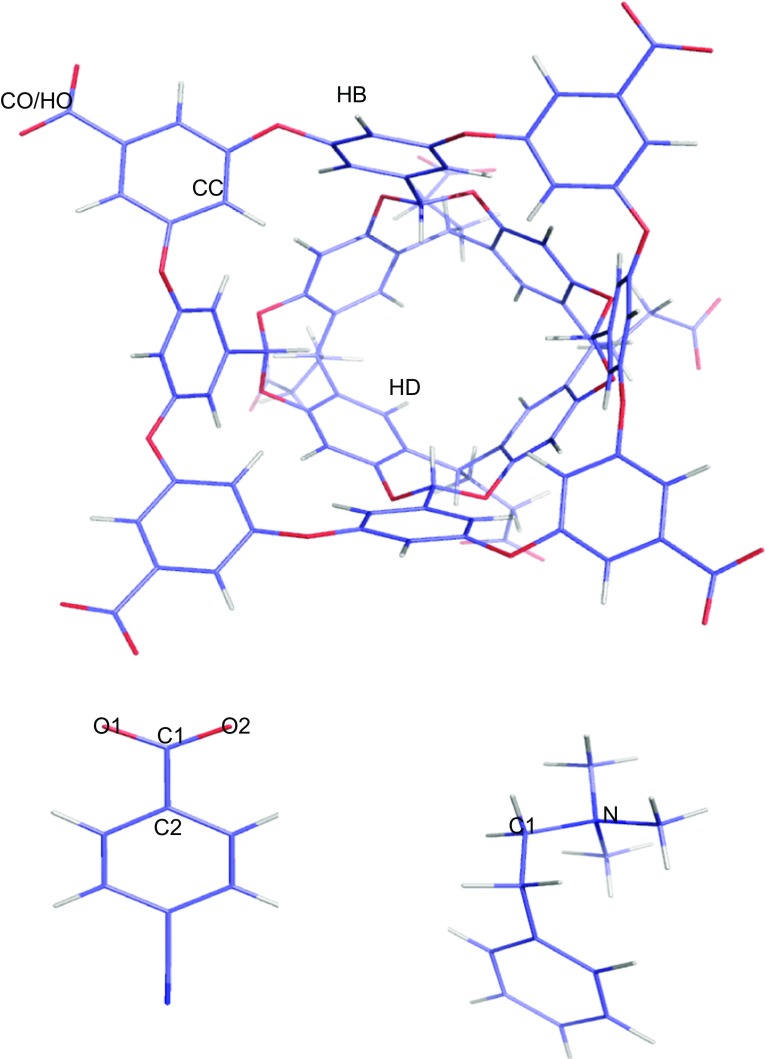
Atom names used in the geometry descriptions

In order to analyse the structures of the host–guest systems, we employed the following geometric measures (the atom names used in the descriptions are shown in Fig. 3 and the measures are illustrated in Figure S1 in the Supplementary material):

- *r*_Dm_ measures how deep the guest is inside the host and is defined as the closest distance between the average of the coordinates of the four HD atoms of the host (AD) and any guest atom.
- α_t_ shows the orientation of the ligand inside the host and is defined as the angle between the C1–C2 (G1, G2, G4, and G6) or N–C1 (G3 and G5) vectors and the host AD–AB vectors, where AB is the average coordinate of the four HB atoms.
- *r*_O1_ and *r*_O2_ or *r*_N_ describe how much the guest carboxylate or trimethylammonium group reaches out of the host. They are the distance between the guest O1 and O2 (for G1, G2, G4, and G6) or N (for G3 and G5) atoms and the average plane defined by the four CC atoms. A positive distance indicates that the atom is outside the host.
- Δ*r*_BB_ measures the distortion of the host and is defined as the difference of the distances between two opposite HB atoms on the host.
- *r*_C1_ and *r*_C2_ describe the orientation of the benzoic (OAH and OAM) or benzene (NOH and NOM) groups. They are calculated as the distance between opposite host CO or HO atoms. *r*_Cav_ is the average of these two distances.
- *r*_min1_ and *r*_min2_ are the two shortest distances between the guest carboxyl oxygen atoms (i.e. only for guests G1, G2, G4, and G6) and a hydrogen atom of the host. They indicate whether there are any CH–O hydrogen bonds.

### Quality estimates

The quality of the binding-affinity estimates compared to experimental data [84] was measured using the mean absolute deviation after removal of the systematic error (i.e. the mean signed deviation; MADtr), the correlation coefficient (*R* ^2^), and Kendall’s rank correlation coefficient (τ). Following the overview article, we employed the NMR data for all complexes, except for G6 and for G4 in OAH, for which ITC data was used [29]. ∆*G*_bind_ of the two experimental data sets differ by 0.3–3.3 kJ/mol (1.5 kJ/mol on average).

## Result and discussion

In this study we have tried to estimate the binding affinities of the twelve octa-acid host–guest systems in the SAMPL5 blind challenge [29]. The octa-acid hosts form a hydrophobic cavity that has been shown to bind various small molecules by hydrophobic interactions inside the cavity [25, 26]. The two variants of the octa-acid cavitand differ in the absence (OAH) or presence (OAM) of four methyl groups on the rim of the cavity, as is shown in Fig. 1. The six guest molecules are shown in Fig. 2. Four of them are negatively charged with a carboxylate group and the other two (G3 and G5) are positively charged with a trimethylammonium group. Three of the hosts contain a benzene ring, one an adamantane group, whereas the other two have linear chains, hexane or pentyne.

Compared to the nine octa-acid OAH–guest systems in the SAMPL4 competition [22], the present ligands shows a much larger diversity, both in the general structure and in the net charge. This make them less suitable for FES calculations of relative binding free energies, which was successfully used by us and other groups in that challenge [22, 27]. Therefore, we decided to instead use the QM approach based on minimised structures, developed by Grimme [30, 31], which was also employed in SAMPL4, giving results of an intermediate quality (*R* ^2^ = 0.6–0.8 and a mean absolute deviation, MAD, of 5–9 kJ/mol) [27, 33].

Using our experience from that study, we aimed at improving the approach in four different aspects:

- Restricting the uncertainty caused by the flexibility of the host molecule by a strict control of the minimisation.
- Reducing the uncertainty caused by the large charge of the host molecules (and also the flexibility) by removing the propionate and benzoic carboxylate groups.
- Testing the effect of a restricted MD sampling.
- Improving the QM method by using the DLPNO–CCSD(T) [34] approach.

The effect of these attempts will be discussed in separate sections.

### Controlled minimisation

Our MD studies of OAH with the nine ligands in the SAMPL4 competition showed that there are two motions that give rise to major variations in the structure of the octa-acid–ligand complexes [27]. The first is a breathing motion of the host, varying the entrance of the cavity from symmetric and circular to elongated and ellipsoidal. It can be described by the ∆*r*_BB_ measure. ∆*r*_BB_ varies by up to 8 Å on a time scale of less than 0.1 ns, but during the minimisation, the distortion is typically frozen into the structure, giving large variations in the minimised structures.

The second motion is in the propionate chains, which have two *sp* ^3^-hybridised dihedrals with three minima of similar energies. Unfortunately, this rotation is rather slow, on the 1–10 ns scale, so very long simulations are needed to sample all possible conformations. Therefore, again different conformations are frozen into the minimised structures and owing to the negatively charged carboxylate group at the end of the chains, the conformations may significantly affect the binding affinities.

To minimise the effect of these two movements we decided to control the minimisations much stricter than in the SAMPL4 challenge. We assumed that none of the ligands should have any certain preference of the host distortion or the propionate conformations. Therefore, we tried to get structures for all ligands that are as similar as possible with regard to the host distortion and the propionate conformations. This was done by first optimising the OAH and OAM hosts with enforced four-fold symmetry. Then, the guests were inserted as symmetric as possible and the structure was carefully optimised in order keep the geometry close to the starting point.

At this stage, we also had to decide how to perform the optimisation. In SAMPL4, we used three different approaches [27]: The optimisation was performed either in a vacuum, in a COSMO continuum solvent (with a dielectric constant of 80), or in the same COSMO solvent, but with four explicit water molecules forming hydrogen bonds to the carboxylate group of the ligand (present in all nine ligands). The three methods gave some systematic variations in the obtained structures, especially regarding the orientation of the benzoate and propionate groups and how far the ligand reached out of the host. However, somewhat unexpectedly, the vacuum structures gave the most stable binding energies, especially if relaxed interaction energies were considered, probably because the strong electrostatic repulsion between the propionate carboxylate groups in vacuum gave them a similar conformation in all structures. Therefore, we decided to use vacuum-optimised structures also in the present investigation (but the other two methods were also tested for the MD snapshots, see below).

**Fig. 4:**
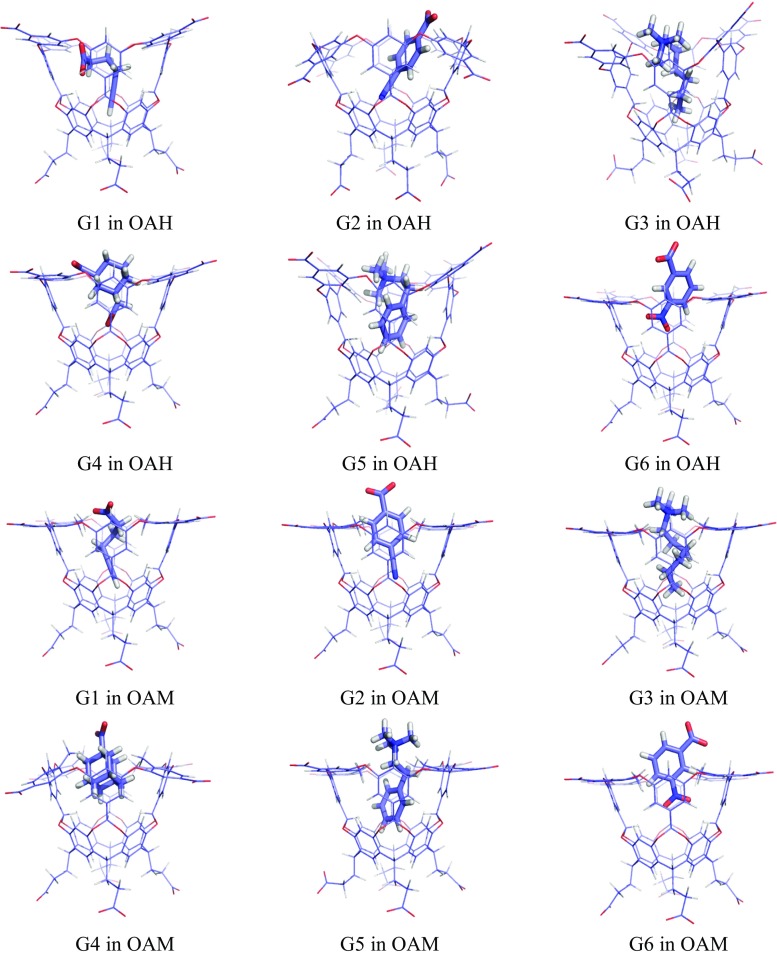
Structures of the OAH (*top*) and OAM (*bottom*) complexes optimised at the TPSS-D3/def2-SV(P) level

The results of these TPSS-D3/def2-SV(P) optimisations are shown in Fig. 4 and described in Table 1. The guests bind inside the host, with at least one of the carboxylate or amine nitrogen atoms above the rim, about ~1 Å over the average plane of the four CB atoms (*r*_O1_ and *r*_O2_ or *r*_N_ in Table 1). However, G6 is only partly buried with most of the benzene ring above the rim of the host. The controlled-minimisation approach was partly successful: For the OAH host, guests G1, G4, and G6 gave nearly symmetric structures with ∆*r*_BB_ < 0.6 Å. However, the two positively charged hosts (G3 and G5) gave more distorted hosts (Fig. 4). The results for the OAM hosts were similar, although the distortion was slightly larger for the negatively charged guest molecules, ∆*r*_BB_ < 1.2 Å.

**Table 1:** Geometric measures for G1–G6 bound to the four hosts after optimisation at TPSS-D3/def2-SV(P) level

Guest	Host	*r*_Dm_	α_t_	*r*_N_/*r*_O1_	*r*_O2_	Δ*r*_BB_	*r*_Cav_	*r*_min1_	*r*_min2_
G1	OAH	2.6	82.8	−2.0	0.2	0.5	19.6	1.9	2.7
	NOH	3.0	76.5	−2.2	−0.2	6.3	17.6	2.4	3.8
	OAM	3.1	47.1	0.6	1.5	0.5	20.1	2.5	2.6
	NOM	2.5	83.3	−0.8	−1.3	2.2	17.8	2.1	2.2
G2	OAH	3.2	47.9	1.6	0.0	2.9	18.3	2.1	2.1
	NOH	4.0	39.9	1.5	1.4	3.8	17.6	1.9	1.9
	OAM	4.1	12.6	3.3	2.8	1.0	20.1	3.0	3.6
	NOM	2.6	1.0	1.8	1.9	3.6	17.7	2.5	2.6
G3	OAH	2.7	50.2	0.6		0.9	17.8		
	NOH	2.7	47.6	1.2		5.4	17.6		
	OAM	2.8	48.4	1.5		0.1	20.0		
	NOM	3.1	51.7	1.7		7.6	17.5		
G4	OAH	4.5	65.3	1.2	1.3	0.5	19.8	2.0	2.0
	NOH	4.1	65.2	0.8	0.9	1.9	17.9	1.9	1.9
	OAM	5.2	13.6	3.5	3.0	1.2	19.9	2.7	3.2
	NOM	5.4	61.7	2.5	1.7	0.3	17.2	2.3	2.4
G5	OAH	2.3	115.8	1.2		0.4	18.2		
	NOH	3.6	26.2	1.0		2.3	17.7		
	OAM	3.3	27.7	2.2		1.1	19.9		
	NOM	3.5	27.8	2.5		1.9	17.9		
G6	OAH	5.9	35.3	4.6	3.1	0.6	20.0	4.4	5.0
	NOH	3.8	59.1	1.1	−0.3	6.6	17.4	2.0	2.2
	OAM	6.0	49.3	2.7	4.3	0.5	20.1	2.7	3.0
	NOM	7.4	53.9	1.6	2.9	1.8	17.8	2.3	2.5

Distances are in Å, angles in degrees

Strangely, G2 did not bind inside the OAH host with the standard method of optimisation. We had to run the optimisation in a COSMO continuum solvent with a dielectric constant of 80 to obtain a bound structure. The results presented in this paper are obtained with that structure. Likewise, G4 tended to dissociate from the OAM host in the initial optimisations, but this could be solved by using carefully designed starting structures.

### Neutralised hosts

The −8 charge of the OAH and OAM hosts gives rise to very large solvation free energies (up to −6620 kJ/mol). These are to a large extent cancelled when the difference in solvation energy between the complex, the host, and the guest are calculated (to around –1580 kJ/mol) and then further cancelled when combined with the QM binding energy, which includes the electrostatic repulsion or attraction between the host and the negatively or positively charged ligands, respectively, giving a net binding free energy of −10 to −39 kJ/mol. Therefore, both the continuum-solvation and the QM methods need to be extremely accurate to give a proper accuracy of the final estimates.

To avoid these problems, we recently suggested and showed for the SAMPL4 ligands that the both the benzoic carboxylate group and the full propionate chain can be replaced by hydrogen atoms (giving the NOH and NOM hosts in Fig. 1), without changing the relative binding free energies of the ligands by more than 2 kJ/mol [72]. In fact, as shown in Table S1 in the Supplementary material, the 2 kJ/mol difference comes mainly from the propionate ligand. Another advantage with the removal of the propionate groups is that the problem with the conformational sampling of these groups is also avoided.

**Fig. 5:**
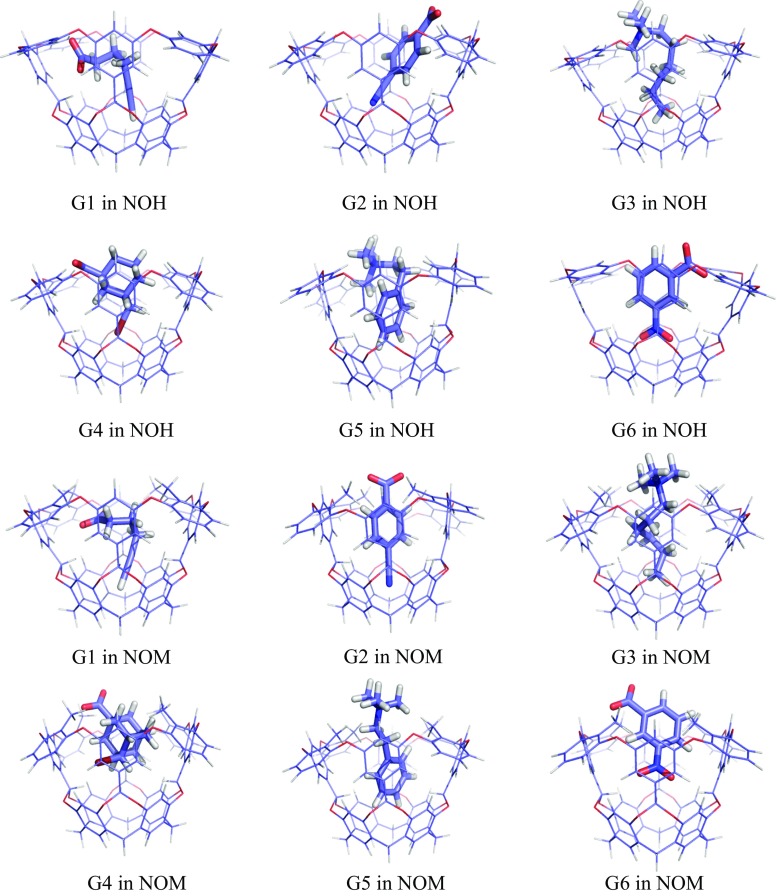
Structures of the NOH (*top*) and NOM (*bottom*) complexes optimised at the TPSS-D3/def2-SV(P) level

Structures of the complexes of all ligands with the neutralised NOH and NOM hosts are shown in Fig. 5 and are described in Table 1. Unfortunately, these structures became much more distorted than the corresponding OAH and OAM structures, with ∆*r*_BB_ = 0.3–7.6 Å, and a large variation among the ligands. It is not clear why the neutral hosts gave such a large distortion, but perhaps the repulsion of the carboxylate groups kept the charged host complexes symmetric in the vacuum optimisation.

### Binding free energies

Next, we estimated the binding affinities for each host–guest system employing the four energy terms in Eq. 1 [27, 30, 33]. The energy terms and the net binding free energies are listed in Table 2. Unless otherwise stated, we discuss only rigid binding energies, i.e. energies obtained with the geometry of the ligand and the host taken from that in the complex, because this gave better and more stable energies in the SAMPL4 calculations [27].

**Table 2:** Calculated energy components and absolute binding free energies (kJ/mol) obtained from TPSS-D3/def2-SV(P) optimised structures

Host	Guest	∆*E*_QM_	∆*E*_disp_	∆*G*_solv_	∆*G*_therm_	Δ*G*_tot_	∆*E*_Grlx_	Δ*G*_tot,Grlx_	Δ*G*_tot,rlx_	Δ*G*_tot,CC_
OAH	G1	948.0	−112.5	−873.4	77.9	40.0	−22.7	62.8	57.4	
	G2	1017.0	−121.0	−957.8	71.3	9.5	−1.9	11.4	−5.1	
	G3	−1062.1	−168.5	1155.9	96.2	21.4	−37.2	58.6	43.1	
	G4	973.3	−154.4	−906.3	101.1	13.7	0.0	13.7	4.8	
	G5	−1043.3	−173.8	1136.7	90.1	9.7	−7.6	17.3	18.7	
	G6	909.5	−71.4	−899.1	72.2	11.2	−1.7	12.9	1.6	
NOH	G1	−72.4	−127.1	186.7	75.6	62.7	−16.4	79.1	67.6	83.9
	G2	−19.9	−122.1	101.4	80.2	39.5	−0.9	40.4	63.2	46.2
	G3	10.2	−149.7	73.4	88.0	21.9	−35.1	57.0	59.4	57.4
	G4	−26.1	−163.4	129.2	81.1	20.8	0.0	20.8	19.7	19.7
	G5	18.3	−165.0	68.7	81.3	3.2	−4.6	7.8	19.4	15.1
	G6	−14.3	−143.0	112.3	79.1	34.1	−2.3	36.4	56.6	49.7
OAM	G1	953.0	−104.4	−888.3	87.8	48.1	−6.4	54.5	39.8	
	G2	950.1	−99.3	−904.5	77.1	23.4	−0.3	23.7	37.7	
	G3	−955.6	−163.6	1065.1	90.2	36.1	−24.0	60.1	57.1	
	G4	1013.3	−173.8	−888.1	95.1	46.5	0.0	46.5	60.4	
	G5	−949.2	−170.1	1121.3	83.7	85.8	−7.4	93.2	18.9	
	G6	911.2	−74.8	−890.6	92.2	38.1	−7.8	45.9	29.0	
NOM	G1	−63.7	−123.0	197.0	81.7	91.9	−13.5	105.4	98.7	103.3
	G2	2.7	−131.0	89.3	78.4	39.4	−3.8	43.2	79.5	50.6
	G3	30.0	−153.5	66.3	96.9	39.7	−23.1	62.8	71.2	53.7
	G4	28.5	−168.0	93.9	98.5	52.9	−5.8	58.7	83.4	79.1
	G5	27.7	−147.4	56.8	86.0	23.1	−4.6	27.7	36.8	37.0
	G6	−29.1	−94.3	81.1	80.0	37.7	−1.6	39.3	44.2	30.4

The energy terms are described in Eq. 1

The first term is the single-point vacuum TPSS/def2-QZVP’ binding energy. For the OAH and OAM hosts, this term is large, owing to the electrostatic interaction between the host with a −8 charge and the ligands with a −1 or +1 charge, 910–1017 or −949 to −1062 kJ/mol, respectively. The energy is ~ 100 kJ/mol less negative in OAM than in OAH for the positively charged ligands, whereas for the other guests there is no consistent difference. For the neutralised hosts, ∆*E*_QM_ is much smaller, −72 to +30 kJ/mol. It is 9–20 kJ/mol more positive in NOM than in NOH for the positively charged ligands, but again without any consistent trend for the negatively charged ligands.

∆*E*_QM_ is more than compensated by the COSMO-RS solvation energy. ∆*G*_solv_ is very large for the OAH and OAM hosts, −873 to −958 kJ/mol for the negatively charged guests but 1056–1165 kJ/mol for G3 and G5. Consequently, the sum of these two terms is always positive, 10–172 kJ/mol, largest for G5 in OAM and lowest for G6 in OAH. For the neutralised hosts, ∆*G*_solv_ is always positive, 57–197 kJ/mol, without any clear difference between the guests with different charges. The sum of the two terms is also always positive, 52–133 kJ/mol.

The dispersion energy is always negative, −71 to −174 kJ/mol. It is more negative for the two positively charged ligand and the bulky G4 ligand than for the other three ligands. It is typically least negative for G6, reflecting that G6 does not bind deeply in the host (except in NOH; cf. Figs. 4, 5). Interestingly, ∆*E*_disp_ is always more negative in the neutralised hosts for the negatively charged ligands, but the opposite is true for the positively charged ligand.

The thermal corrections vary only slightly among the various systems. They are always positive, 71–101 kJ/mol, reflecting the loss of translational and rotational entropy when the guest molecule binds to the host. There are no consistent differences for the various hosts and no correlation between the results obtained with the charged and neutralised hosts.

Summing the four terms gives the net binding free energy, ∆*G*_tot_. Somewhat disappointingly, it is positive for all ligands, 3–92 kJ/mol. There is no consistent difference between the positively or negatively charged ligands. However, ∆*G*_tot_ is more positive for the truncated hosts than for the fully charged ones (by 0–30 kJ/mol), except for G6. There is no correlation between the results obtained for the charged and neutralised hosts, *R* ^2^ = 0.1.

**Fig. 6:**
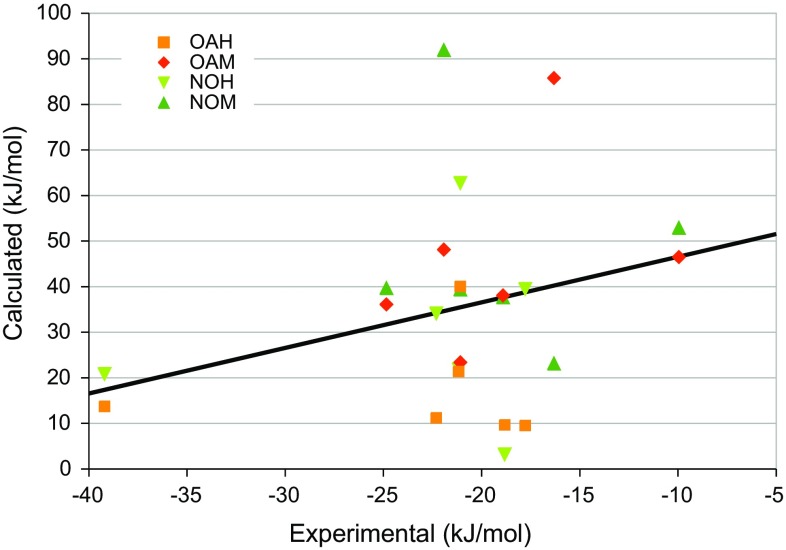
Comparison of the rigid binding free energies for the fully charged and the neutralised hosts [84]. The *line* shows the experimental affinity plus 56 kJ/mol (the average difference between the calculated and experimental affinities)

Unfortunately, the calculated binding free energies show no correlation (*R* ^2^ = 0.0–0.1) with the experimental affinities [84], as can be seen in Fig. 6 and Table 3. Kendall’s τ is also low and varying, −0.3 to 0.3. The MADtr is rather large, 11–16 kJ/mol, slightly larger for the neutralised hosts than for the fully charged hosts. Errors over 20 kJ/mol are obtained for G1 in all hosts except OAM and G5 in all hosts except OAH. The other ligands give errors lower than 14 kJ/mol, except G2 in OAM. The large errors for G1 is most likely caused by the fact that it binds with the carboxylate group inside all hosts except OAM (Figs. 4 and 5), in contrast to explicitly solvated MD simulations, in which G1 binds the carboxylate group in the solvent (Figure S2). This will be further examined below.

**Table 3:** Quality measures (compared to experimental data [84]) of the three total binding free energies obtained with TPSS-D3/def2-SV(P) minimised structures (TPSS), MD sampled structures (MD), and structures minimised with HF-3c in a COSMO continuum solvent without (Cos) or with four water molecules

		TPSS				MD	Cos	Wat
		∆*G*_tot_	∆*G*_tot,Grlx_	∆*G*_tot,rlx_	∆*G*_tot,CC_	∆*G*_tot_	∆*G*_tot_	∆*G*_tot_
MADtr	OAH	11.2	19.3	18.8				
	NOH	14.2	17.0	15.0	16.5	14.8	16.1	11.3
	OAM	13.9	17.5	12.0				
	NOM	15.9	21.6	19.8	19.8	16.6	16.0	11.8
*R* ^2^	OAH	0.01	0.05	0.05				
	NOH	0.03	0.08	0.30	0.15	0.01	0.00	0.04
	OAM	0.14	0.01	0.01				
	NOM	−0.02	−0.09	−0.02	0.00	−0.08	−0.06	−0.05
τ	OAH	−0.33	−0.07	−0.07				
	NOH	0.20	0.20	0.33	0.07	0.20	0.07	0.20
	OAM	0.33	−0.07	−0.33				
	NOM	−0.33	−0.47	−0.20	−0.20	−0.20	−0.20	−0.20

A negative value of *R* ^2^ indicates that *R* is negative

The ligand relaxation energy (∆*E*_Grlx_ in Table 2) is less than 8 kJ/mol for most of the ligands, except G1 and G3 with the linear chains (up to 37 kJ/mol). Including this energy (Δ*G*_tot,Grlx_ in Tables 2, 3) of course makes the binding free energy even more positive. This does not change the correlation significantly, but MADtr increases for all hosts. Thus, the results are not improved by including the ligand relaxation energy. If all energy terms are calculated for the fully relaxed host and guest molecules (∆*G*_tot,rlx_ in Tables 2, 3), the correlation improves slightly for NOH, *R* ^2^ = 0.3, and MADtr improves compared to ∆*G*_tot,rlx_, but it is still worse than ∆*G*_tot_ for all hosts except OAM, 12–20 kJ/mol. Therefore, we will only discuss the rigid results in the following.

Compared to the corresponding results for the SAMPL4 octa-acid challenge [27], the present calculations give appreciably worse results (*R* ^2^ = 0.0–0.1 and MADtr = 11–15 kJ/mol, compared to 0.6–0.8 and 5–9 kJ/mol). In particular, all the present binding affinities are positive, whereas this was the case only for one ligand in the SAMPL4 set. The only difference between the two sets of calculations is the use of the HF-3c method for the ∆*G*_therm_ term, rather than MM. This term is 71–101 kJ/mol for the SAMPL5 complexes, but it was only 44–57 kJ/mol for the SAMPL4 MM results. For the Bz complex in SAMPL4 [27], the difference in the ∆*G*_therm_ calculated with MM and HF-3c is 30 kJ/mol, indicating a significant difference in the results obtained with the two methods. The difference comes entirely from the vibrational part and it is dominated by the entropy contribution (showing that it is caused mainly by the low-frequency vibrations), but both the enthalpy and zero-point energy parts are also significantly different (4–6 kJ/mol), although the enthalpy part counteracts the other two contributions. Test calculations indicated that the scale factor of the HF-3c frequencies (0.86) had only minor influence on the results (a scale factor of 1.0 changed the results by only 3 kJ/mol). Sure and Grimme recommended the HF-3c method to obtain vibrational frequencies for host–guest binding affinities [13], but in this case this method seems to give significantly worse results than MM.

Therefore, we recalculated the ∆*G*_therm_ term for all the OAH and OAM complexes with MM (no scaling of the frequencies). The results are shown in Table S2 in the Supplementary material. It can be seen that ∆*G*_therm_ in general is larger when calculated with HF-3c, by 11 kJ/mol on average, but the difference is varying −2 to 32 kJ/mol. In particular, even when calculated with MM, ∆*G*_therm_ is larger for the present ligands, 58–98 kJ/mol, than for the SAMPL4 ligands. This indicates that the difference in the thermostatistical corrections between the SAMPL4 and SAMPL5 sets comes primarily from differing properties of the ligands, rather than from the change in the method used to calculate the vibrational frequencies.

The difference in the rigid guest energies between the methylated and non-methylated hosts indicate how the methylation affects the guest binding. The ∆*E*_QM_ energy differences are rather small for most of the ligands (up to 6 kJ/mol), but 12–16 kJ/mol for G3 in both hosts and G1 in the charged hosts. For both ligands, the methylated hosts give the smaller distortion of the guest. This indicates that we may have studied suboptimal structures of the flexible G1 and G3 guests in the less crowed unmethylated hosts, i.e. a sampling problem.

We also calculated binding free energies using PBE/def2-QZVP’ calculations and dispersion parameters for PBE (because this approach gave better results than TPSS for other host–guest systems [31]). The PBE/def2-QZVP’ binding energies were 30 kJ/mol more favourable than the TPSS energies on average, but this was compensated by the dispersion energies, so that the net binding free energies differed by −4 to +11 kJ/mol (3 kJ/mol on average, i.e. PBE gave a slightly weaker binding). This did not change the results significantly and therefore only TPSS results will be discussed in the following.

### Coupled-cluster calculations

Finally, we recalculated the QM energies with the more accurate DLPNO–CCSD(T) method. This approach can provide CCSD(T) energies with extrapolations to a complete basis set using def2-TZVPP and def2-QZVPP calculations even for the present complexes of up to 184 atoms. The calculations were based on the TPSS-D3/def2-SV(P) optimised structures (only neutralised hosts) and the DLPNO–CCSD(T) rigid interaction energies were combined with the DFT solvation energies and the HF-3c thermostatistical corrections (∆*G*_solv_ and ∆*G*_therm_ in Table 2) to give net binding free energies. The results are given in the last column (Δ*G*_tot,CC_) in Table 2.

The raw DLPNO–CCSD(T) rigid interaction energies differ from the TPSS-D3/def2-QZVP’ ∆*E*_QM_ + ∆*E*_disp_ energies by 1–36 kJ/mol. In general, the CCSD(T) energies are somewhat more positive (by 13 kJ/mol on average); the TPSS-D3 energies are more positive only for G4 in NOH and G6 in NOM (by 1 and 7 kJ/mol, respectively). The largest differences are found for G1 and G3 in NOH and for G4 in NOM (21, 36, and 26 kJ/mol), whereas for the other ligands, the difference is up to 16 kJ/mol. The DLPNO–CCSD(T) energies are reasonably converged with respect to the basis set: A basis-set extrapolation based on the smaller def2-SVP and def2-TZVPP basis set gave results that differed by less than 8 kJ/mol (4 kJ/mol on average).

Unfortunately, the DLPNO–CCSD(T) calculations did not improve the results compared to experiments (Table 3): There is still no correlation between the experimental and calculated results, and the MADtr increased slightly compared to the TPSS-D3 energies (16–20 kJ/mol). Based on benchmark calculations and previous studies of intermolecular interactions with DLPNO–CCSD(T), it is reasonable to expect that the results are within 4 kJ/mol of that of canonical CCSD(T) [85–87]. Since the latter method is known to be accurate for such interaction energies between organic closed-shell molecules, we believe that the electronic energies from the coupled-cluster calculations are close to chemical accuracy (4 kJ/mol relative to the exact solution of the electronic Schrödinger equation at fixed geometry). While this is certainly a methodological achievement, these results highlight the importance of the other terms that enter the free energy and demonstrates that the accuracy of the calculated binding free energies is not limited by the energy calculations.

### MD sampled structures

One of the largest problems with the present approach is the use of single minimised structures. For large flexible molecules, it is hard to find the global minimum and it is possible that several conformations have a low energy, all contributing to the binding free energy. We have already taken several precautions to reduce this problem, using rigid interaction energies, keeping all complexes as symmetric and similar as possible, and removing the flexible propionate groups.

As an alternative and more general approach, we also tested to use a set of structures sampled from MD simulations. For each host–guest system, we run a 10 ns MD simulation of the explicitly solvated complex, started from the TPSS/def2-SV(P) structures. From these, we took ten regularly spaced snapshots, which were minimised and energies were then calculated using the same four energy terms in Eq. 2 as for the original minimised structures. To save time, the calculations were performed on the neutralised NOH and NOM hosts and the minimisations were performed at the HF-3c level. Moreover, test calculations showed that the ∆*G*_therm_ term did not change significantly for the various structures, so we used the same value (in Table 2) for all snapshots. Only rigid interaction energies were considered. Of course, this is a rather primitive approach to include some effects of the conformational flexibility of the complexes and more accurate approaches exist [88, 89]. However, it will give a first indication of the importance of structure sampling within the present optimisation approach.

**Fig. 7:**
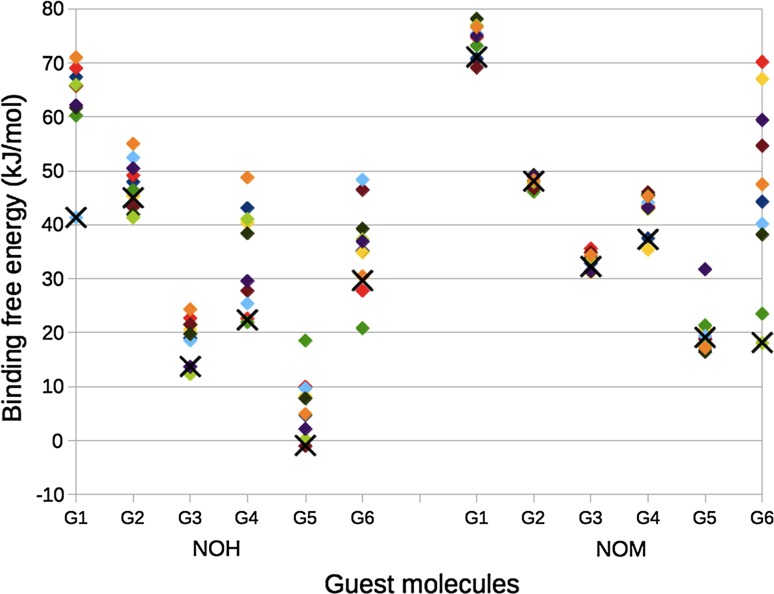
Binding free energies (∆*G*_tot_ in kJ/mol) from the MD sampling. Each *diamond symbol* represents the results from on of the ten snapshots. The *black crosses* are the Boltzmann-weighted averages

**Fig. 8:**
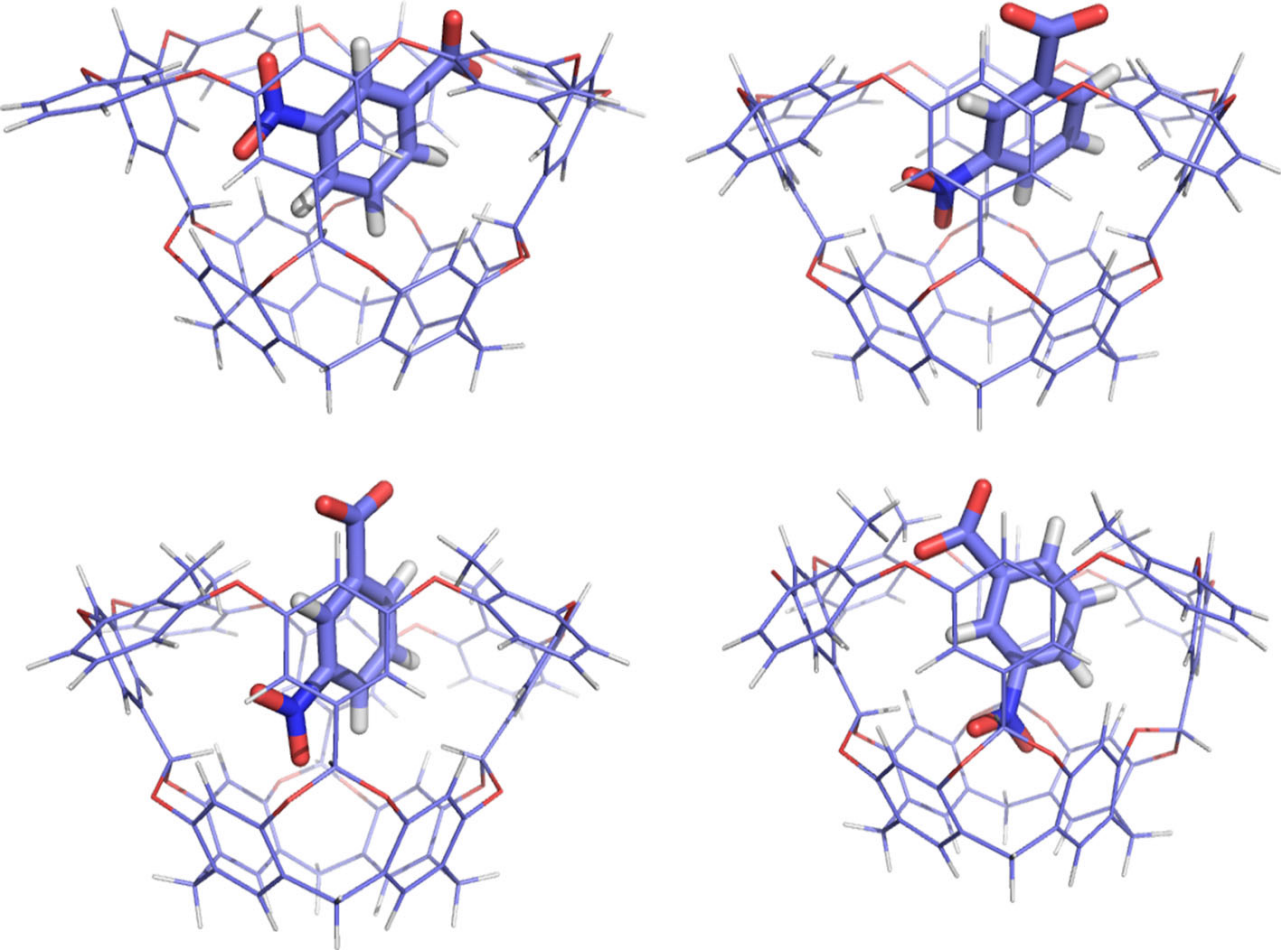
Varying orientations of G6 inside NOH (*top*) and NOM (*bottom*) after minimisation of the MD snapshots

The calculated binding free energies (∆*G*_tot_) for these ten sets of calculations are shown in Fig. 7. It can be seen that variation of the free energies is quite restricted for most of the ligands, with ranges of 3–30 kJ/mol, implying standard errors for pure averages of 0.3–3.0 kJ/mol. Only G6 in NOM gives a larger and even spread of 50 kJ/mol (standard error of 5 kJ/mol). This is caused by the fact that G6 has two bulky groups in meta positions, which can bind in several orientations (cf. Fig. 8). Otherwise, the NOM host always gives a smaller variation in the binding free energies than the NOH host, most likely because the methyl groups restrict the number of possible binding modes. The bimodal variation of G4 in NOH is caused by the fact that the carboxylate atoms do not point straight upwards in the optimised structures but instead form a varying number of hydrogen bonds with the HC atoms of the host in different snapshots: The more positive binding free energies are obtained when the guest forms one hydrogen bond, whereas less positive free energies are obtained when it forms two hydrogen bonds.

Owing to the change in the energy function (MM in the simulations, but the final energies are calculated at the TPSS/def2-QZVP’ level) pure averages should not be used when evaluating the binding affinities. Instead, the snapshots should be Boltzmann-weighted, giving higher weights to the structures with the lowest energies of the complexes. These Boltzmann-weighted averages are shown as crosses in Fig. 7 and it can be seen that in general, the most favourable complexes also give the most favourable binding energies, reducing the influence of high-energy outliers. On the other hand, it strongly increases the importance of the structures with the most favourable binding free energy and in four cases the final binding affinities are determined from one single structure (G1, G3, and G5 for NOH and G6 in NOM). For G1 in NOH, this structure is a low-energy outlier, viz. the only structure in which the carboxylate group of G1 is not buried inside the host, giving it a ~50 kJ/mol more favourable ∆*G*_solv_ than the other structures.

**Fig. 9:**
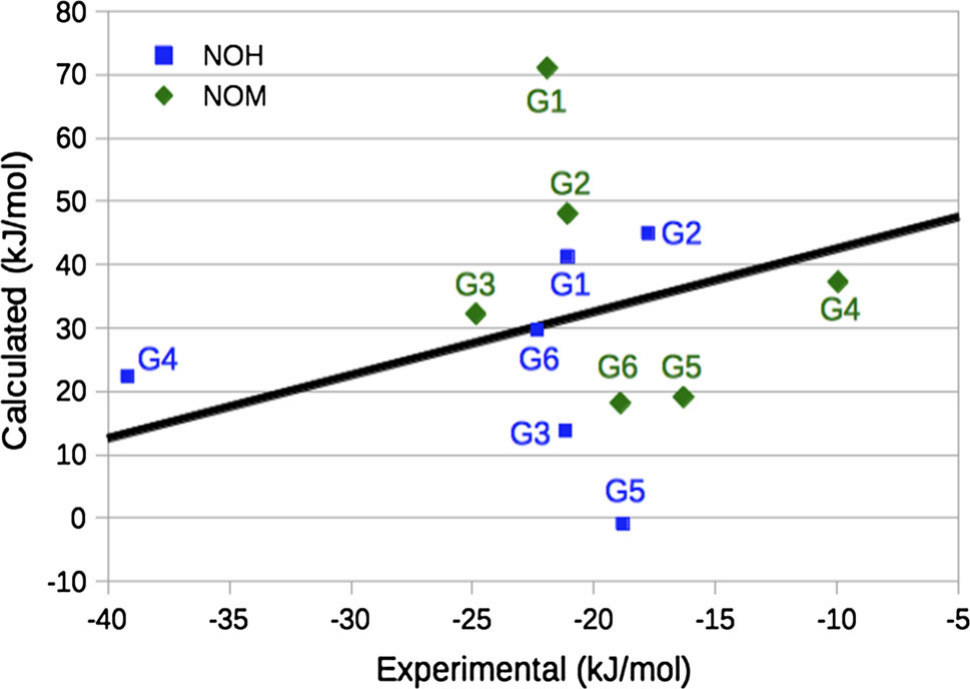
Comparison of the Boltzmann-averaged binding free energies from the MD snapshots and experiments [84]. The *line* shows the experimental affinity plus 52 kJ/mol (the average difference between the calculated and experimental affinities)

Figure 9 shows how the Boltzmann-averaged binding affinities compare to experiments [84]. It can be seen that the agreement is still quite poor. Both hosts give a rather high MADtr, 15–17 kJ/mol (Table 3), and all binding free energies are still 49–57 kJ/mol too positive. Moreover, the correlation to the experimental affinities is poor (*R* ^2^ = 0.0–0.1). However, the reason for this is mainly some outliers: First, the positively charged G3 and G5 give too favourable binding energies (except G3 in NOM). This seems to be a problem with the solvation model or at least poor cancellation of errors in the solvation free energy for the guests of different net charge. In fact, intuitively G3 and G5 should have even more favourable binding affinities, owing to the attraction between the strongly negatively charged octa-acid host and the positively charge ligands (a simple unit-charge water-screened Coulomb model gives an attraction of ~25 kJ/mol in the complex), which is missing for the neutralised hosts. However, a comparison of the results for the two hosts in Table 2 do not give any indication of any need of such a correction and it would only make the results deviate more from experiments.

Second, G1 in NOM has a much too positive binding affinity. In fact, there is a similar problem of G1 in NOH, but a single low-energy outlier provided a reasonable binding free energy after Boltzmann averaging (Fig. 7). As discussed above, this is related to the vacuum optimisations, which give structures with the carboxylate groups buried too deeply in the host (Figs. 4, 5). Third, G6 in NOM also has a somewhat too favourable binding, but this is mainly caused by the Boltzmann-averaging—the pure average is instead above the expected correlation line.

Inspired by the problem with G1, we tried to improve the structures by performing the optimisation in a COSMO continuum solvent with a dielectric constant of 80, either without or with four water molecules interacting with the carboxylate or trimethylammonium group of the ligands (as was also done in our SAMPL4 study [27]). The water molecules were deleted before the binding energies were calculated and the ∆*G*_therm_ term was not recalculated. To enhance the chance to obtain reasonable structures, the optimisations were started from one random snapshot from the MD simulations. The optimisation was performed at the HF-3c level of theory.

**Fig. 10:**
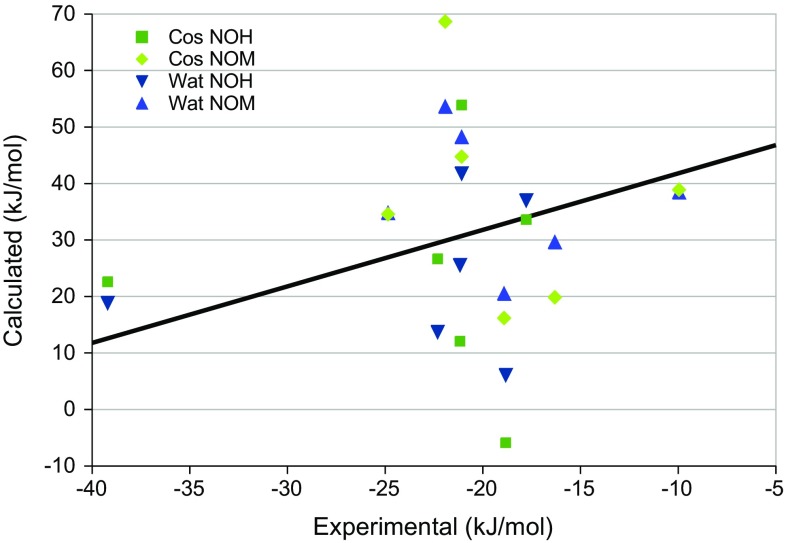
Comparison of the experimental binding free energies [84] and the calculated results based on one MD snapshot optimised with HF-3c in a COSMO continuum solvent without (Cos) or with four explicit water molecules (Wat). The line shows the experimental affinity plus 52 kJ/mol (the average difference between the calculated and experimental affinities)

As will be discussed more in the next section, this approach improved several of the structures in that the charged group became less buried in the hosts. This had significant effects also on the binding free energies. The calculated binding free energies are shown in Fig. 10. It can be seen that the two approaches still give essentially no correlation to the experimental data. However, the MADtr are reduced to 11–12 kJ/mol with the explicit water molecules (cf. Table 3), primarily because the results were improved for G1 and G5. However, the largest errors are still obtained for these two ligands (10–22 kJ/mol). G6 also gives a large error 11–17 kJ/mol, whereas G3 gives good results. The net binding affinities are listed in Table S4 in the supplementary material.

### Comparison of structures

We have obtained structures with five different approaches: snapshots from MD simulation at the MM level, the latter snapshots optimised by HF-3c in a vacuum, one MD snapshot optimised with HF-3c in a COSMO continuum solvent without or with four explicit water molecules, and built symmetrised structures optimised with TPSS/def2-SV(P) in a vacuum. The TPSS optimisation involved both the fully charged hosts and the neutralised models, whereas the other four sets involved only the neutralised hosts. For all five sets, hosts both with and without the methyl groups have been studied. In this section, we will analyse the differences between the structures obtained with the various methods. The TPSS structures were described in Table 1 and shown in Figs. 4 and 5, whereas the other structures are described in Table 4.

**Table 4:** Geometric measures of complexes obtained from the MD snapshots with different methods: average value over the ten MD snapshots (MM), average value over the ten MD snapshots optimised in vacuum with HF-3c (Vac), or one MD snapshot optimised with HF-3c in a COSMO continuum solvent without (Cos) or with four explicit water molecules (Wat)

Host	Guest	Method	*r*_Dm_	α_t_	*r*_N_/*r*_O1_	*r*_O2_	Δ*r*_BB_	*r*_Cav_	*r*_min1_	*r*_min2_
NOH	G1	MM	3.0	42.1	1.3	1.5	1.1	17.8	3.3	3.9
		Vac	2.2	90.9	−1.3	−0.1	3.0	17.7	1.8	2.9
		Cos	2.2	80.5	−0.2	1.8	2.6	17.7	1.8	2.8
		Wat	2.4	57.8	1.2	1.2	0.3	17.7	2.3	4.4
	G2	MM	4.4	32.2	2.7	2.4	1.9	17.7	2.5	3.1
		Vac	3.7	36.7	−1.3	−0.1	3.8	17.4	1.9	1.9
		Cos	3.7	34.3	−0.2	1.8	3.7	17.4	2.0	2.0
		Wat	3.7	32.9	1.2	1.2	2.8	17.4	2.1	2.1
	G3	MM	3.4	24.1	3.1		1.2	17.6		
		Vac	3.0	30.0	2.7		2.8	17.5		
		Cos	3.1	19.0	2.9		0.9	17.4		
		Wat	2.9	20.3	2.7		2.1	17.6		
	G4	MM	5.8	33.6	3.0	2.9	1.0	17.9	3.5	4.4
		Vac	4.6	64.2	1.5	1.3	0.9	17.6	1.7	2.7
		Cos	4.0	62.7	0.0	0.4	0.5	17.6	1.8	3.0
		Wat	4.1	59.8	0.1	0.9	0.1	17.5	2.0	3.3
	G5	MM	3.9	31.8	2.6		1.3	17.9		
		Vac	3.5	30.8	2.3		3.1	17.5		
		Cos	3.5	28.4	2.2		0.7	17.4		
		Wat	3.5	29.7	2.2		0.7	17.4		
	G6	MM	5.1	64.9	2.6	2.7	4.5	17.4	3.0	3.9
		Vac	4.4	68.4	0.7	1.4	3.6	17.4	1.8	3.0
		Cos	4.7	57.5	1.6	1.9	4.3	17.3	2.0	3.6
		Wat	4.3	50.6	1.8	1.8	5.7	17.2	2.4	4.1
NOM	G1	MM	3.1	40.9	2.2	2.4	1.5	17.8	3.0	3.1
		Vac	1.9	64.7	0.2	1.0	2.7	17.5	2.0	2.1
		Cos	2.1	54.8	0.9	2.4	1.2	17.5	2.1	2.1
		Wat	2.4	41.8	2.1	0.9	1.4	17.5	2.5	2.8
	G2	MM	4.3	12.6	3.4	3.2	1.8	17.7	3.3	3.6
		Vac	2.5	14.1	2.0	1.9	0.3	17.6	2.6	2.9
		Cos	2.6	11.7	1.8	2.3		17.5	2.8	2.9
		Wat	2.6	14.7	1.8	2.2		17.5	2.6	3.1
	G3	MM	3.5	36.2	3.5		1.7	17.7		
		Vac	2.8	33.5	2.9		4.3	17.4		
		Cos	3.0	28.2	2.6		2.8	17.5		
		Wat	2.9	30.2	2.7		2.3	17.5		
	G4	MM	5.4	19.7	2.9	2.8	0.7	17.2	2.9	3.4
		Vac	5.2	27.9	2.6	2.6	1.3	16.9	2.4	3.3
		Cos	5.1	25.7	2.6	2.8	1.7	16.9	2.2	3.5
		Wat	4.9	26.8	2.3	2.9	1.8	16.8	2.3	3.6
	G5	MM	4.5	21.7	3.5		1.6	17.6		
		Vac	3.7	21.9	2.6		4.8	17.3		
		Cos	3.6	20.0	2.8		3.7	17.4		
		Wat	3.7	13.7	2.8		1.2	17.7		
	G6	MM	5.0	22.4	3.2	2.5	2.0	17.5	3.1	3.4
		Vac	3.8	40.0	2.2	0.5	1.0	17.3	2.2	2.5
		Cos	4.5	16.4	3.2	1.9	1.2	17.5	2.8	3.7
		Wat	3.5	28.8	2.4	1.0	0.2	17.3	3.1	3.2

Results with both the NOH and NOM hosts are given

Owing to the charges of both the ligands and the hosts, it is likely that the MD simulations in explicit solvent give the most realistic structures. The largest difference between the MD structures and the vacuum-optimised structures is that the charged groups of the ligands are reaching further out into the solvent in the MD structures. This is most clearly seen from *r*_O1/N_, which is positive and rather large for all MD structures, 1.3–3.5 Å, whereas it is always smaller and often negative for the optimised structures. The effect is larger for the negatively charged ligands than for G3 and G5. It can partly be cured by optimising in COSMO with or without explicit water molecules, but the results are varying and the same approach does not always give the best structures. There is often a large difference between the TPSS results obtained with the full or neutralised hosts, reflecting the problem of using only a single structure.

The MD structures also give larger *r*_min_ distances (2.5–3.5 Å) than the minimised structures, reflecting that the carboxylate atoms form hydrogen bonds with water rather than with the host CH atoms. Again, this can be improved by the use of explicit water molecules in the optimisation, but the improvement is only partial and for some complexes, the difference is still 1.5 Å. The ligand is typically also deeper buried in the host in the optimised structures than in the MD structures, as is illustrated by the *r*_Dm_ distance (3.0–5.8 Å for the latter structures), but the variation is quite large between the various complexes.

For some complexes, there is also a large difference in the orientation of the guest in the host between the MD and optimised structures, indicated by the α_t_ tilt angle. The difference is particularly large for G1 in both hosts (41–42° in MD compared to 65–91° in the minimised structures) and G4 in OAH or NOH (34° compared to 63–65°). For the former, the results are improved with COSMO and explicit water molecules, especially for NOM, but not for G4. On the other hand, there is no consistent difference in the distortion of the host or the orientation of the benzyl groups between the MD and optimised structures.

For many of the OAH and OAM structures optimised with TPSS in vacuum, the benzoate groups are tilted upwards or outwards (cf. Fig. 4), whereas in the MD structures, these groups are tilted downwards. This is an effect of the missing solvation in the vacuum-optimised structures: If the structures are instead optimised in the COSMO continuum solvent, the benzoate groups tilt downwards, as was observed in our SAMPL4 study [27]. Likewise, if the benzoate groups are deleted, as in the NOH and NOM structures, the remaining benzene rings tilt downwards. However, the tilt of these groups seem to have little influence on the guest binding energies, considering that structures optimised in vacuum or with COSMO solvation gave similar binding energies in SAMPL4 [27].

There are few general differences between the structures obtained for hosts with or without the methyl groups. For all ligands, except G3, the α_t_ tilt angle is smaller for the methylated hosts. This is most pronounced for G6, for which the difference is 42° in the MD structures, whereas it is 20° for G2 and around 10° for G4 and G5. Structures obtained with the other approaches typically show qualitatively similar differences, but with larger variations, especially for G1 and G6. This indicates that the methyl groups force the ligand to bind more upright.

The methylated hosts also in general give a larger *r*_O1/N_ distance (by 0.4–0.9 Å for the MD structures), with few exceptions (the most important is the MD structures of G4, for which NOH gives a 0.1 Å smaller distance on average). This indicates that methyl groups make the ligands protrude somewhat more from the host. However, the ligands still reach to a similar depth into the hosts, with a difference in *r*_Dm_ ranging from −0.4 Å (G4) to 0.6 Å (G5) for the MD structures.

There are quite extensive variations within the snapshots taken from the MD simulations. In particular, α_t_ varies by 16–52° in the various simulations (more with NOM than with NOH) and the ∆*r*_BB_ distortion by 1–5 Å. The *r*_Dm_ and *r*_O1/N_ distances show a smaller variation of 0.6–2.9 and 0.9–3.6 Å, respectively. In the HF-3c structures optimised from the MD snapshots, the variation can both be reduced and enhanced. For example, all HF-3c structures of G2 in NOM are essentially identical, whereas they show an extensive variation in the MD snapshots (e.g. a variation in α_t_ of 3–31° and 14–15° before and after the optimisation). On the other hand, the variation of ∆*r*_BB_ is only 2 Å for the MD structures of G5 in NOH, but 7 Å for the HF-3c structures started from these snapshots.

G6 shows two low-energy conformations in the optimised structures in both hosts, characterised of α_t_ = 45–57° or 97–102° in NOH and 17–18° or 45–49° in NOM. It represents two orientations of the nitro group inside the host, as can be seen in Fig. 8. There is also an extensive variation of the distortion of the host and in how deep the ligand binds in the host, but both variables are independent of the change in the ligand conformation. All this variation in the structure gives rise to the extensive variation in the binding energies seen in Fig. 7.

### Submitted results

Three sets of data (relative binding free energies) were submitted for each host: DFT energies based on the TPSS-D3/def2-SV(P) structures with either the OAH/OAM or the NOH/NOM hosts, as well as DLPNO–CCSD(T) energies, based on the latter structures (called DFT-charged, DFT-neutral, and CCSD(T)-neutral, respectively, in the overview article [29]). The other binding affinities discussed in this article were not finished at the time of the submission. Moreover, G2 had dissociated from OAH and G4 bound outside the cavity with the carboxylate group directed inwards the cavity for both the OAM and NOM hosts. All energies included the ∆*E*_Grlx_ term (i.e. they were Δ*G*_tot,Grlx_), except those for OAH, which were fully relaxed free energies (∆*G*_tot,rlx_; the rigid host energies were not finished before submission). Finally, the DLPNO–CCSD(T) energies were based only on the def2-SVP/TZVPP basis-set extrapolation and the 7.9 kJ/mol correction for the change in reference state was omitted. Finally, some of the solvation energies were incorrect. The submitted data are shown in Table S5 in the Supplementary material.

The submitted data gave much better correlation to the experimental results than the data presented in Table 3 (*R* ^2^ = 0.1–0.5), but worse MADtr, 20–43 kJ/mol). The results in this article provide the correct data for the current methods. It is clear that these methods are not competitive compared to the best methods for this test case, giving *R* ^2^ = 0.7–0.8 and MADtr = 4–6 kJ/mol (but very few methods gave both good *R* ^2^ and MADtr and also good results for both hosts).

## Conclusions

As a part of the SAMPL5 host–guest competition, we have tried to estimate the free energy for the binding of six small, but diverse ligands to two variants of the octa-acid cavitand. Our aim was to test and improve a method, originally suggested by Grimme [30, 31], employing DFT calculations with large basis sets, empirical dispersion corrections (DFT-D3) [32, 40], continuum estimates of the solvation free energy [48, 49], as well as enthalpy and entropy corrections from vibrational frequencies [30, 54], all estimated from single minimised DFT structures. This approach was used for the same host in the SAMPL4 competition by both Grimme and us, giving results of intermediate quality [27, 33].

Based on those calculations, we tried to improve the calculations in four ways. First, we reduced the effect of the flexibility of the host by a strict control of the host molecules, keeping them as symmetric and similar as possible during the geometry optimisations. In particular, we controlled the breathing motion of the host and the conformation of the propionate groups. Moreover, we employed geometry optimisation in vacuum, which enhance the repulsion between the propionate and benzoate groups, thereby increasing the symmetry of the complexes [27]. We also calculated rigid interaction energies, using the geometry of the host and guest from the complex also for the isolated moieties, because this gave somewhat more stable energies (but it was less important than in our previous study [27]). Thereby, we could obtain quite symmetric complexes for all the negatively charged ligands, but for the two ligands with the bulky trimethylammonium group, the complexes were still quite distorted.

Second, we performed calculations also on host molecules for which we had removed all the propionate and benzoate groups, thereby both reducing the flexibility and deleting the large negative charge, which gives rise to very large QM and solvation energy terms that need to cancel very accurately to give reliable final results. Test calculations on the SAMPL4 ligands with FES methods showed that these charged groups had only minimal effects (<2 kJ/mol) on the relative binding free energies [72]. Unfortunately, the structures of the neutralised host were more distorted than those of the charged host, probably owing to the repulsion of the charged groups in the vacuum optimisation.

Third, we tested to perform a restricted conformational sampling by employing ten snapshots from a MD simulation. The results (Fig. 7) showed a rather limited variation in the binding free energies calculated from the various snapshots for most of the ligands, except G6. The variation was typically smaller in the methylated host, owing to a more restricted binding site.

Fourth, we tried to improve the QM energies with the DLPNO–CCSD(T) approach [34]. In SAMPL4, we employed local LCCSD(T0) calculations [90], but we needed to use fractionation methods [91] for the large complexes [27, 92]. With the DLPNO–CCSD(T) approach, such approximations could be avoided, and no strong deterioration of the results was observed, as in the previous studies. However, the results were not improved, indicating that the performance is not limited by the accuracy of the QM method.

In spite of all these attempts to improve the results, the calculated binding free energies compared poorly with the experimental results [84], giving no correlation, systematically too positive binding energies by ~50 kJ/mol, and a MADtr of 11–16 kJ/mol. This is much worse than the corresponding results for the SAMPL4 ligands, for which we had essentially no systematic error, *R* ^2^ = 0.6–0.8, and MAD = 5–9 kJ/mol [27]. We have identified at least four possible sources of this poor performance:

- The use of vacuum-optimised structures is a major problem, giving structures that differ significantly from those obtained in MD simulations in explicit solvent. The problem can be partly reduced by performing the optimisations in a continuum solvent with a few explicit water molecules around the charged group of the ligands (MADtr = 11–12 kJ/mol). However, the structures are still not fully satisfactorily for all ligands.
- Conformational sampling is still a problem (especially in combination with the optimised structures) for some of the ligands, especially G6. It can be solved by using more snapshots from MD, but the optimisation method is still a problem.
- There are indications that the COSMO-RS method has problems to provide solvation energies that are comparable for both the negatively and positively charged ligands. In particular, the positively charged G3 and G5 ligands give large errors.
- Thermostatistical corrections from HF-3c structures are more positive than those obtained by MM methods (as in our SAMPL4 calculations). These corrections seem to be the prime cause of the systematic error of the present calculations.

In conclusion, it seems currently hard to obtain accurate ligand-binding affinities with QM methods and minimised structures. In particular, the QM methods are not competitive with FES methods, based on MM sampling. The problem is not the DFT-D or DLPNO–CCSD(T) energy functions, but rather the sampling, geometry optimisation, as well as the solvation and thermostatistical corrections. The octa-acid system with its large negative charge seems to pose a large problem for the QM approach and this is further enhanced by ligands of a varying net charge.

An alternative approach to obtain binding free energies with QM methods is to use free-energy simulations with reference-potential methods (i.e. performing the MD simulations at the MM level and then performing perturbations or reweighting from MM to QM) [14, 72, 93, 94]. Unfortunately, the overlap between the MM and QM potential surfaces are so poor that very many QM calculations are needed to obtain converged results, e.g. 720,000 QM calculations for each of the SAMPL4 octa-acid ligands to obtain a precision of 1 kJ/mol [72]. This is ~4000 times more than an approach with single minimised structures, showing that such approach may remain competitive even with quite extensive sampling, provided that the problems with the optimisation, solvation, and thermal corrections can be solved.

## Electronic supplementary material

Below is the link to the electronic supplementary material.

Supplementary material 1 (PDF 448 kb)

## References

[1] Gohlke H, Klebe G (2002). Angew Chem Int Ed.

[2] Jorgensen WL (2009). Acc Chem Res.

[3] Zhou H-X, Gilson MK (2009). Chem Rev.

[4] Michel J, Essex JW (2010). J Comput Aided Mol Des.

[5] Christ CD, Mark AE, van Gunsteren WF (2010). J Comput Chem.

[6] Wereszczynski J, McCammon JA (2012). Quart Rev Biophys.

[7] Söderhjelm P, Ryde U (2009). J Phys Chem A.

[8] Cavalli A, Carloni P, Recanatini M (2006). Chem Rev.

[9] Raha K, Peters MB, Wang B, Yu N, Wollacott AM, Westerhoff LM, Merz KM (2007). Drug Discov Today.

[10] Söderhjelm P, Kongsted J, Genheden S, Ryde U (2010). Interdiscip Sci Comput Life Sci.

[11] Söderhjelm P, Genheden S, Ryde U, Gohlke H (2012). Methods and principles in medicinal chemistry.

[12] Antony J, Grimme S (2012). J Comput Chem.

[13] Sure R, Grimme S (2015). J Chem Theory Comput.

[14] Ryde U, Söderhjelm P (2016). Ligand-binding affinity estimates supported by quantum-mechanical methods. Chem Rev.

[15] Houk KN, Leach AG, Kim SP, Zhang XY (2003). Angew Chem Int Ed.

[16] Moghaddam S, Inoue Y, Gilson MK (2009). J Am Chem Soc.

[17] Monroe JI, Shirts MR (2014). J Comput Aided Mol Des.

[18] Hsiao Y-W, Söderhjelm P (2014). J Comput Aided Mol Des.

[19] Muddana HS, Yin J, Sapra NV, Fenley AT, Gilson MK (2014). J Comput Aided Mol Des.

[20] Jensen JH (2015). Phys Chem Chem Phys.

[21] Muddana HS, Varnado CD, Bielawski CW, Urbach AR, Isaacs L, Geballe MT, Gilson MK (2012). J Comput Aided Mol Des.

[22] Muddana HS, Fenley AT, Mobley DL, Gilson MK (2014). J Comput Aided Mol Des.

[23] Naïm M, Bhat S, Rankin KN, Dennis S, Chowdhury SF, Siddiqi I, Drabik P, Sulea T, Bayly CI, Jakalian A (2007). J Chem Inf Model.

[24] Hogues H, Sulea T, Purisima E (2014). J Comput Aided Mol Des.

[25] Sun H, Gibb CLD, Gibb BC (2008). Supramol Chem.

[26] Gibb CLD, Gibb BC (2009). Tetrahedron.

[27] Mikulskis P, Cioloboc D, Andrejic M, Khare S, Brorsson J, Genheden S, Mata RA, Söderhjelm P, Ryde U (2014). J Comput Aided Mol Des.

[28] Gibb CL, Gibb BC (2004). J Am Chem Soc.

[29] Yin J, Henriksen NM, Slochower DR, Chiu MW, Mobley DL, Gilson MK (2016) J Comp-Aided Mol Des (in press) 10.1007/s10822-016-9974-4 PMC5241188 27658802

[30] Grimme S (2012). Chem Eur J.

[31] Antony J, Sure R, Grimme S (2015). Chem Commun.

[32] Grimme S (2011). WIREs Comput Mol Sci.

[33] Sure R, Antony J, Grimme S (2014). J Phys Chem B.

[34] Riplinger C, Neese F (2013). J Chem Phys.

[35] Gan H, Benjamin CJ, Gibb BC (2011). J Am Chem Soc.

[36] Ahlrichs R, Bär M, Häser M, Horn H, Kölmel C (1989). Chem Phys Lett.

[37] Treutler O, Ahlrichs RJ (1995). Chem Phys.

[38] Tao J, Perdew JP, Staroverov VN, Scuseria GE (2003). Phys Rev Lett.

[39] Weigend F, Ahlrichs R (2005). Phys Chem Chem Phys.

[40] Grimme S, Antony J, Ehrlich S, Krieg H (2010). J Chem Phys.

[41] Perdew JP, Burke K, Ernzerhof M (1996). Phys Rev Lett.

[42] http://www.thch.uni-bonn.de/tc/index.php?section=downloads&subsection=getd3

[43] Eichkorn K, Treutler O, Öhm H, Häser M, Ahlrichs R (1995). Chem Phys Lett.

[44] Eichkorn K, Weigend F, Treutler O, Ahlrichs R (1997). Theor Chem Acc.

[45] Sierka M, Hogekamp A, Ahlrichs R (2003). J Chem Phys.

[46] Klamt A, Schüürmann G (1994). J Chem Soc Perkin Trans.

[47] Schäfer A, Klamt A, Sattel D, Lohrenz JCW, Eckert F (2000). Phys Chem Chem Phys.

[48] Klamt A (1995). J Phys Chem.

[49] Eckert F, Klamt A (2002). AIChE J.

[50] Eckert F, Klamt A (2010). COSMOtherm, Version C30, Release 1301.

[51] Becke AD (1988). Phys Rev A.

[52] Perdew JP (1986). Phys Rev B.

[53] Schäfer A, Horn H, Ahlrichs R (1992). J Chem Phys.

[54] Jensen F (1999). Introduction to computational chemistry.

[55] Sure R, Grimme S (2013). J Comput Chem.

[56] Genheden S, Ryde U (2015). Expert Opinion Drug Discov.

[57] Riplinger C, Neese F (2013). J Chem Phys.

[58] Riplinger C, Sandhoefer B, Hansen A, Neese F (2013). J Chem Phys.

[59] Riplinger C, Pinski P, Becker U, Valeev EF, Neese F (2016). J Chem Phys.

[60] Neese F (2012). Wires Comput Mol Sci.

[61] Weigend F (2006). Phys Chem Chem Phys.

[62] van Wüllen C (1998). J Chem Phys.

[63] Pantazis DA, Chen XY, Landis CR, Neese F (2008). J Chem Theory Comput.

[64] Zheng J, Xu X, Truhlar DG (2010). Theor Chem Acc.

[65] Boys SF, Bernardi F (1970). Mol Phys.

[66] Neese F, Valeev EF (2011). J Chem Theory Comput.

[67] Liakos DG, Sparta M, Kesharwani MK, Martin JML, Neese F (2015). J Chem Theory Comput.

[68] Sparta M, Marius R, Peter P, Ute B, Christoph R, Neese F (2016) in preparation

[69] Horn HW, Swope WC, Pitera JW, Madura JD, Dick TJ, Hura GL, Head-Gordon T (2004). J Chem Phys.

[70] Case DA, Berryman JT, Betz RM, Cerutti DS, Cheatham TE, Darden TA, Duke RE, Giese TJ, Gohlke H, Goetz AW, Homeyer N, Izadi S, Janowski P, Kaus J, Kovalenko A, Lee TS, LeGrand S, Li P, Luchko T, Luo R, Madej B, Merz KM, Monard G, Needham P, Nguyen H, Nguyen HT, Omelyan I, Onufriev A, Roe DR, Roitberg A, Salomon-Ferrer R, Simmerling CL, Smith W, Swails J, Walker RC, Wang J, Wolf RM, Wu X, York DM, Kollman PA (2014). AMBER 14.

[71] Wang JM, Wolf RM, Caldwell KW, Kollman PA, Case DA (2004). J Comput Chem.

[72] Olsson MA, Söderhjelm P, Ryde U (2016). J Comput Chem.

[73] Dewar MJS, Zoebisch EG, Healy EF, Stewart JJP (1985). J Am Chem Soc.

[74] Hehre WJ, Ditchfield R, Pople JA (1972). J Chem Phys.

[75] Besler BH, Merz KM, Kollman PA (1990). J Comput Chem.

[76] Frisch MJ, Trucks GW, Schlegel HB, Scuseria GE, Robb MA, Cheeseman JR, Scalmani G, Barone V, Mennucci B, Petersson GA, Nakatsuji H, Caricato M, Li X, Hratchian HP, Izmaylov AF, Bloino J, Zheng G, Sonnenberg JL, Hada M, Ehara M, Toyota K, Fukuda R, Hasegawa J, Ishida M, Nakajima T, Honda Y, Kitao O, Nakai H, Vreven T, Montgomery JA, Peralta JE, Ogliaro F, Bearpark M, Heyd JJ, Brothers E, Kudin KN, Staroverov VN, Kobayashi R, Normand J, Raghavachari K, Rendell A, Burant JC, Iyengar SS, Tomasi J, Cossi M, Rega N, Millam JM, Klene M, Knox JE, Cross JB, Bakken V, Adamo C, Jaramillo J, Gomperts R, Stratmann RE, Yazyev O, Austin AJ, Cammi R, Pomelli C, Ochterski JW, Martin RL, Morokuma K, Zakrzewski VG, Voth GA, Salvador P, Dannenberg JJ, Dapprich S, Daniels AD, Farkas O, Foresman JB, Ortiz JV, Cioslowski J, Fox DJ (2009). Gaussian 09, revision A02.

[77] Bayly CI, Cieplak P, Cornell WD, Kollman PA (1993). J Phys Chem.

[78] Seminario JM (1996). Int J Quant Chem.

[79] Nilsson K, Lecerof D, Sigfridsson E, Ryde U (2003). Acta Crystallogr D.

[80] Wang JM, Wolf RM, Caldwell KW, Kollman PA, Case DA (2004). J Comput Chem.

[81] Wu XW, Brooks BR (2003). Chem Phys Lett.

[82] Berendsen HJC, Postma JPM, van Gunsteren WF, Dinola A, Haak JR (1984). J Chem Phys.

[83] Darden T, York D, Pedersen L (1993). J Chem Phys.

[84] Rustenburg AS, Dancer J, Lin B, Ortwine DF, Mobley DL, Chodera JD (2016) J Comput Aided Chem Des (submitted) 10.1007/s10822-016-9971-7 PMC5209288 27718028

[85] Pinski P, Riplinger C, Valeev EF, Neese F (2015). J Chem Phys.

[86] Liakos DG, Sparta M, Kesharwani MK, Martin JML, Neese F (2015). J Chem Theory Comput.

[87] Liakos DG, Neese F (2015). J Chem Theory Comput.

[88] Chang CE, Potter MJ, Gilson MK (2003). J Phys Chem B.

[89] Cecchini M, Krivov SV, Spichty M, Karplus M (2009). J Phys Chem B.

[90] Hampel C, Werner H-J (1996). J Chem Phys.

[91] Söderhjelm P, Ryde U (2009). J Phys Chem A.

[92] Andrejic M, Ryde U, Mata R, Söderhjelm P (2014). Chem Phys Chem.

[93] Luzhkov V, Warshel A (1992). J Comput Chem.

[94] König G, Boresch S (2011). J Comput Chem.

