# Supplementary material for: Binding free energies in the SAMPL5 octa-acid host–guest challenge calculated with DFT-D3 and CCSD(T)
Source: J Comput Aided Mol Des. 2016 Sep 6;31(1):87–106. doi: 10.1007/s10822-016-9957-5 (PMC5239813; doi:10.1007/s10822-016-9957-5)

Supplementary material

Binding free energies in the SAMPL5 octa-acid host–guest challenge calculated with DFT-D3 and CCSD(T)

Octav Caldararu,[a] Martin A. Olsson,[a] Christoph Riplinger,[b] Frank Neese,[b] Ulf Ryde [a]*

[a] Department of Theoretical Chemistry, Lund University, Chemical Centre, P. O. Box 124, SE-221 00 Lund, Sweden

[b] Max-Planck-Institut für Chemische Energiekonversion, Stiftstraße 34-36, 45470 Mülheim an der Ruhr, Germany

Correspondence to Ulf Ryde, E-mail: Ulf.Ryde@teokem.lu.se,

Tel: +46 – 46 2224502, Fax: +46 – 46 2228648

2016-08-23

Table S1. Relative binding free energies for the nine ligand involved in the SAMPL4 blind test competition [22], calculated with FES using the OAH [27], NOH [72], and HOH host molecules.

	OAH		HOH		NOH	
MeBz→Bz	15.94	±0.05	15.62	±0.04	15.72	±0.02
EtBz→MeBz	1.02	±0.08	2.81	±0.06	3.28	±0.02
pClBz→Bz	19.06	±0.09	18.97	±0.04	18.60	±0.02
mClBz→Bz	6.11	±0.12	6.80	±0.05	6.81	±0.03
Hx→Bz	13.14	±0.32	14.74	±0.09	15.60	±0.05
MeHx→Hx	15.35	+0.15	14.84	±0.03	14.81	±0.01
Hx→Pen	7.48	±0.73	8.19	±0.07	8.33	±0.04
Hep→Hx	5.50	±0.66	7.44	±0.09	7.35	±0.07

Table S2. Comparison of the ΔG_{therm} term calculated with HF-3c (also shown in Table 2) and MM.

Host	Guest	HF-3c	MM
OAH	G1	77.9	75.1
	G2	71.3	72.9
	G3	96.2	72.8
	G4	101.1	77.5
	G5	90.1	57.7
	G6	72.2	71.2
OAM	G1	87.8	65.1
	G2	77.1	77.6
	G3	90.2	80.2
	G4	95.1	97.5
	G5	83.7	73.1
	G6	92.2	79.8

Table S3. PBE results, using fully relaxed structures.

Host	Guest	ΔE_{QM}	ΔE_{disp}	$\Delta G_{tot, rlx}$
OAH	G1	965.4	-84.8	54.1
	G2	1365.3	-123.6	2.0
	G3	-1005.5	-154.3	42.9
	G4	953.5	-115.5	1.0
	G5	-1022.0	-148.1	22.1
	G6	905.4	-53.3	3.1
NOH	G1	-62.9	-100.1	65.3
	G2	-19.1	-94.9	52.9
	G3	24.2	-124.8	56.0
	G4	-56.0	-123.6	18.0
	G5	6.7	-129.7	19.0
	G6	-11.0	-112.9	54.0
OAM	G1	941.3	-79.2	30.7
	G2	950.5	-74.4	25.4
	G3	-960.5	-136.9	48.6
	G4	1067.7	-121.1	41.5
	G5	-954.1	-131.9	14.8
	G6	909.3	-56.9	22.3
NOM	G1	-58.5	-100.3	93.8
	G2	14.4	-105.8	69.1
	G3	37.3	-131.6	67.3
	G4	17.8	-128.3	70.4
	G5	16.1	-111.3	34.6
	G6	-42.1	-75.1	35.0

Table S4. Calculated energy components and absolute binding free energies (kJ/mol) obtained from the Boltzmann-averaged MD snapshots (MD) and one MD snapshot optimised with HF-3c in a COSMO continuum solvent without (Cos) or with four explicit water molecules (Wat). The energy terms are described in Eqn. 1.

		MD	Cos				Wat			
Host	Guest	ΔG_{tot}	ΔE_{QM}	ΔE_{disp}	ΔG_{solv}	ΔG_{tot}	ΔE_{QM}	ΔE_{disp}	ΔG_{solv}	ΔG_{tot}
NOH	G1	41.4	-36.4	-105.5	120.1	53.9	-35.3	-92.6	94.0	41.8
	G2	45.0	-6.7	-127.6	87.8	33.6	-9.5	-123.7	90.0	37.0
	G3	13.7	6.8	-112.9	30.1	12.1	6.5	-116.5	47.5	25.5
	G4	22.4	3.3	-170.2	108.4	22.6	1.9	-163.5	99.3	18.8
	G5	-0.9	11.9	-134.2	35.2	-5.9	11.3	-135.7	49.2	6.1
	G6	29.7	-6.8	-116.0	70.4	26.7	-2.6	-118.6	55.8	13.7
NOM	G1	71.1	-36.8	-102.9	126.7	68.7	-28.2	-103.1	103.2	53.6
	G2	48.1	11.1	-128.1	83.3	44.7	13.2	-130.7	87.3	48.2
	G3	32.3	15.6	-120.9	43.0	34.5	19.2	-122.2	40.9	34.8
	G4	37.3	52.3	-181.1	69.1	38.9	54.5	-184.5	69.9	38.4
	G5	19.1	23.1	-138.2	48.9	19.8	22.4	-124.2	45.4	29.6
	G6	18.2	-5.3	-109.3	50.8	16.2	11.7	-135.0	63.8	20.5

Table S5. Submitted data and experimental results (ΔG_{bind} in kJ/mol relative to G1). In addition, R^2 and MADtr compared to experiments are given, omitting G1. As discussed in the section "Submitted results" in the main article, these data contain several errors and suboptimal structures.

Set		Set 1	Set 2	Set 3	
Method		DFT	DFT	CCSD(T)	Exp.
Host		Neutral	Charged	Neutral	
OAH	G1	0.0	0.0	0.0	0.0
or	G2	-15.9	-4.6	-28.9	3.1
NOH	G3	7.1	-14.2	15.1	1.8
	G4	-58.2	-52.7	-87.4	-17.4
	G5	-20.9	-38.5	-37.2	4.6
	G6	-42.7	-72.0	-54.8	0.4
	R^2	0.48	0.14	0.47	
	MADtr	26.7	34.9	42.5	
OAM	G1	0.0	0.0	0.0	0.0
or	G2	-39.3	-7.9	-48.1	0.9
NOM	G3	-46.9	5.4	-42.3	-2.1
	G4	23.0	54.0	22.2	12.5
	G5	-18.8	38.5	-23.0	6.1
	G6	-66.1	-7.5	-82.8	3.7
	R^2	0.47	0.41	0.41	
	MADtr	38.0	20.3	42.9	

Table S6. Amber topology files for NOH, NOM, and the six guest molecules, as well as the Amber parameter file for the missing dihedral angle of guest G2.

noh.in

```
    0    0    2
oa0.dat
OA0, with RESP charges, 5/3-14
oa0.dat
  OA0  INT     1
 CHANGE OMIT DU   BEG
   0.00000
   1  DUMM  DU    M     0.0000  0.0000   .0000   .0000
   2  DUMM  DU    M     0.0000  1.0000   .0000   .0000
   3  DUMM  DU    M     1.0000  1.0000   .0000   .0000
   4  C55   ca    M    -1.5220  3.2080 -4.2130 -0.137304
   5  H28   ha    E    -0.9350  3.9470 -3.6740  0.077201
   6  C42   ca    M    -2.4280  2.4090 -3.5740 -0.011167
   7  C56   ca    S    -3.2280  1.6060 -4.2980 -0.137304
   8  H29   ha    E    -4.1010  1.1070 -3.8840  0.077201
   9  C9    c3    M    -2.6020  2.4590 -2.0300  0.114807
  10  O3    os    E    -2.3830  3.7840 -1.5580 -0.224878
  11  H19   h2    E    -1.7860  1.8200 -1.6630  0.135169
  12  O1    os    M    -3.8870  1.8990 -1.6480 -0.224878
  13  C3    ca    M    -3.9100  1.1440 -0.5370  0.066965
  14  C1    ca    M    -3.8090 -0.1940 -0.7940 -0.182589
  15  H1    ha    E    -3.7770 -0.4960 -1.8380  0.203172
  16  C4    ca    M    -3.5960 -1.0770  0.2820  0.066965
  17  O2    os    E    -3.5150 -2.4080 -0.0920 -0.224878
  18  C5    ca    M    -3.5760 -0.6530  1.6280  0.062476
  19  C7    c3    3    -3.4220 -1.7270  2.6740 -0.029682
  20  H12   hc    E    -4.0350 -1.4450  4.1080  0.057240
  21  C32   ca    S    -1.9080 -2.1060  2.8250  0.062476
  22  C31   ca    S    -1.0700 -1.3980  3.6830 -0.311116
  23  H56   ha    E    -1.4800 -0.5880  4.2800  0.175692
  24  H4    hc    E    -4.0520 -2.5450  2.3190  0.057240
  25  C2    ca    M    -3.6140  0.7180  1.8500 -0.311116
  26  H2    ha    E    -3.5190  1.1430  2.8460  0.175692
  27  C6    ca    M    -3.8150  1.6430  0.7650  0.062476
  28  C8    c3    M    -3.5870  3.1110  1.0700 -0.029682
  29  H13   hc    E    -4.2140  3.7790  2.3520  0.057240
  30  H3    hc    E    -4.0610  3.5940  0.2130  0.057240
  31  C10   ca    M    -2.1180  3.3410  0.8270  0.062476
  32  C15   ca    S    -1.2000  3.2470  1.8990 -0.311116
  33  H5    ha    E    -1.4830  2.7760  2.8370  0.175692
  34  C14   ca    M    -1.6790  3.8550 -0.3830  0.066965
  35  C17   ca    M    -0.3540  4.2700 -0.5020 -0.182589
  36  H6    ha    E    -0.0230  4.5760 -1.4910  0.203172
  37  C13   ca    M     0.5180  4.3050  0.5390  0.066965
  38  O4    os    S     1.9160  4.5440  0.3420 -0.224878
  39  C34   c3    3     2.7380  3.3120  0.0870  0.114807
  40  C41   ca    B     3.7170  3.4890 -1.0630 -0.011167
  41  C50   ca    S     4.9700  2.8340 -1.1090 -0.137304
  42  H25   ha    E     5.2320  2.2220 -0.2500  0.077201
  43  C51   ca    S     3.2900  4.1570 -2.1870 -0.137304
  44  H26   ha    E     2.3190  4.6440 -2.1600  0.077201
  45  O5    os    E     3.3430  2.9830  1.3450 -0.224878
  46  H18   h2    E     2.1180  2.4680 -0.2510  0.135169
  47  C16   ca    M     0.0690  3.7170  1.7650  0.062476
  48  C18   c3    M     0.9430  3.6670  3.0350 -0.029682
  49  H14   hc    E     0.2660  4.1450  4.3360  0.057240
  50  H7    hc    E     1.7670  4.3580  2.8390  0.057240
  51  C19   ca    M     1.6220  2.2760  2.9700  0.062476
  52  C23   ca    S     1.0540  1.2190  3.7130 -0.311116
  53  H8    ha    E     0.1680  1.3470  4.3280  0.175692
  54  C22   ca    M     2.7970  2.0530  2.2300  0.066965
  55  C25   ca    M     3.4100  0.8020  2.2740 -0.182589
  56  H9    ha    E     4.2770  0.6000  1.6510  0.203172
  57  C21   ca    M     2.9540 -0.1560  3.1840  0.066965
  58  O6    os    S     3.5260 -1.3650  2.9920 -0.224878
  59  C38   c3    3     2.8680 -2.3720  2.1430  0.114807
  60  C40   ca    B     3.7410 -3.0970  1.1560 -0.011167
  61  C45   ca    S     4.6920 -2.4430  0.4170 -0.137304
```

62	H22	ha	E	4.8000	-1.3670	0.5280	0.077201
63	C46	ca	S	3.7040	-4.5050	0.9540	-0.137304
64	H23	ha	E	3.0520	-5.1060	1.5820	0.077201
65	O7	os	E	2.0630	-3.1990	3.0450	-0.224878
66	H20	h2	E	2.1440	-1.8390	1.5090	0.135169
67	C24	ca	M	1.8100	0.0410	3.8860	0.062476
68	C26	c3	M	1.2020	-1.0390	4.7470	-0.029682
69	H15	hc	E	0.5170	-0.7400	6.1700	0.057240
70	H10	hc	E	2.0710	-1.6340	5.0360	0.057240
71	C28	ca	M	0.2870	-1.7610	3.8000	0.062476
72	C30	ca	M	0.7650	-2.8410	3.0580	0.066965
73	C33	ca	M	-0.0940	-3.5100	2.1790	-0.182589
74	H11	ha	E	0.2760	-4.3380	1.5800	0.203172
75	C29	ca	M	-1.3960	-3.1670	2.0210	0.066965
76	O8	os	M	-2.2010	-3.7230	1.1090	-0.224878
77	C39	c3	M	-2.2460	-3.0170	-0.1190	0.114807
78	H21	h2	E	-1.4410	-2.2680	-0.1360	0.135169
79	C43	ca	M	-2.1220	-3.8470	-1.3610	-0.011167
80	C61	ca	S	-2.8770	-3.6480	-2.4770	-0.137304
81	H32	ha	E	-3.5880	-2.8250	-2.4940	0.077201
82	C60	ca	M	-1.1610	-4.8870	-1.5080	-0.137304
83	H31	ha	E	-0.4580	-5.0240	-0.6910	0.077201
84	C63	ca	M	-1.0570	-5.6870	-2.6250	0.170479
85	O16	os	E	-0.0010	-6.5630	-2.7380	-0.295814
86	C59	ca	M	-1.7870	-5.3340	-3.7420	-0.166592
87	H33	ha	E	-1.5690	-5.8270	-4.6860	0.155458
88	C62	ca	M	-2.6680	-4.2930	-3.7170	0.170479
89	O15	os	M	-3.2710	-3.9870	-4.9160	-0.295814
90	C77	ca	M	-4.0610	-2.9060	-5.1440	0.129911
91	C74	ca	S	-3.4630	-1.7490	-5.6080	-0.125196
92	H37	ha	E	-2.3900	-1.5760	-5.6390	0.161643
93	C73	ca	M	-5.3960	-3.1620	-5.3070	-0.070243
94	H39	ha	E	-5.8020	-4.1500	-5.1040	0.152216
95	C76	ca	M	-6.2480	-2.1320	-5.6650	-0.271146
96	H78	ha	E	-7.7260	-2.3950	-5.6970	0.175048
97	C75	ca	M	-5.6930	-0.8190	-5.9550	-0.070243
98	H38	ha	E	-6.2600	0.0840	-6.1660	0.152216
99	C65	ca	M	-4.3320	-0.6840	-5.8620	0.129911
100	O13	os	M	-3.8470	0.5350	-6.3550	-0.295814
101	C57	ca	M	-2.9710	1.3190	-5.6770	0.170479
102	C54	ca	M	-1.8770	1.9370	-6.2420	-0.166592
103	H30	ha	E	-1.5790	1.6560	-7.2490	0.155458
104	C58	ca	M	-1.1840	2.8990	-5.5540	0.170479
105	O14	os	M	-0.2710	3.6030	-6.3370	-0.295814
106	C71	ca	M	0.4830	4.5680	-5.7770	0.129911
107	C70	ca	S	1.8070	4.3690	-5.3040	-0.125196
108	H35	ha	E	2.2100	3.3690	-5.4340	0.161643
109	C68	ca	M	-0.0980	5.8230	-5.7250	-0.070243
110	H36	ha	E	-1.1120	5.9550	-6.0950	0.152216
111	C72	ca	M	0.6450	6.9110	-5.2020	-0.271146
112	H91	ha	E	0.0390	8.2950	-5.3010	0.175048
113	C69	ca	M	1.9590	6.6470	-4.7050	-0.070243
114	H34	ha	E	2.5290	7.4240	-4.2020	0.152216
115	C64	ca	M	2.5180	5.3940	-4.7170	0.129911
116	O11	os	M	3.8140	5.1790	-4.2690	-0.295814
117	C52	ca	M	4.1400	4.3350	-3.2140	0.170479
118	C49	ca	M	5.3730	3.6990	-3.1920	-0.166592
119	H27	ha	E	5.9960	3.9250	-4.0530	0.155458
120	C53	ca	M	5.8060	2.9660	-2.1720	0.170479
121	O12	os	M	6.9260	2.1980	-2.4430	-0.295814
122	C89	ca	M	7.1340	1.1040	-1.6430	0.129911
123	C86	ca	S	6.6520	-0.1000	-2.0110	-0.125196
124	H43	ha	E	6.1020	-0.1680	-2.9460	0.161643
125	C85	ca	M	8.0540	1.1990	-0.5600	-0.070243
126	H45	ha	E	8.4490	2.1800	-0.3090	0.152216
127	C88	ca	M	8.3250	0.1190	0.2670	-0.271146
128	H90	ha	E	8.9880	0.2140	1.6550	0.175048
129	C87	ca	M	7.7270	-1.0940	-0.1070	-0.070243
130	H44	ha	E	7.8830	-1.9790	0.5050	0.152216
131	C67	ca	M	6.8860	-1.2180	-1.1970	0.129911
132	O10	os	M	6.3190	-2.4960	-1.3660	-0.295814
133	C48	ca	M	5.4160	-3.0580	-0.5800	0.170479

```
 134  C44   ca    M      5.1650 -4.3950 -0.9060 -0.166592
 135  H24   ha    E      5.6610 -4.9270 -1.7140  0.155458
 136  C47   ca    M      4.2660 -5.1190 -0.1630  0.170479
 137  O9    os    M      4.1460 -6.4310 -0.3410 -0.295814
 138  C83   ca    M      2.9300 -6.9620 -0.4970  0.129911
 139  C79   ca    M      2.3990 -7.8210  0.3850 -0.070243
 140  H42   ha    E      3.0780 -8.2120  1.1390  0.152216
 141  C82   ca    M      1.1080 -8.2640  0.3810 -0.271146
 142  H84   ha    E      0.6290 -9.2780  1.4400  0.175048
 143  C81   ca    M      0.2650 -7.7360 -0.6370 -0.070243
 144  H41   ha    E     -0.7330 -8.1450 -0.7710  0.152216
 145  C66   ca    M      0.7510 -6.9350 -1.6650  0.129911
 146  C80   ca    M      2.0660 -6.4710 -1.4940 -0.125196
 147  H40   ha    E      2.3850 -5.6980 -2.1880  0.161643


LOOP
   C58  C55
   C57  C56
   C14   O3
    C6   C3
   C39   O2
   C29  C32
   C28  C31
   C16  C15
   C53  C50
   C52  C51
   C22   O5
   C24  C23
   C48  C45
   C47  C46
   C30   O7
   C62  C61
   C66  O16
   C65  C74
   C64  C70
   C67  C86
   C80  C83

IMPROPER
   C58  C42  C55  H28
    C9  C55  C42  C56
   C57  C42  C56  H29
    C1   C6   C3   O1
    C3   C4   C1   H1
    C1   C5   C4   O2
    C7   C4   C5   C2
    C7  C29  C32  C31
   C28  C32  C31  H56
    C5   C6   C2   H2
    C8   C3   C6   C2
    C8  C15  C10  C14
   C10  C16  C15   H5
   C10  C17  C14   O3
   C14  C13  C17   H6
   C17  C16  C13   O4
   C34  C50  C41  C51
   C53  C41  C50  H25
   C52  C41  C51  H26
   C18  C15  C16  C13
   C18  C23  C19  C22
   C19  C24  C23   H8
   C19  C25  C22   O5
   C22  C21  C25   H9
   C25  C24  C21   O6
   C38  C45  C40  C46
   C48  C40  C45  H22
   C47  C40  C46  H23
   C26  C23  C24  C21
   C26  C30  C28  C31
   C28  C33  C30   O7
   C30  C29  C33  H11
```

```
  C33  C32  C29   O8
  C39  C61  C43  C60
  C43  C62  C61  H32
  C43  C63  C60  H31
  C60  C59  C63  O16
  C63  C62  C59  H33
  C61  C59  C62  O15
  C74  C73  C77  O15
  C77  C65  C74  H37
  C77  C76  C73  H39
  H78  C73  C76  C75
  C76  C65  C75  H38
  C74  C75  C65  O13
  C54  C56  C57  O13
  C57  C58  C54  H30
  C54  C55  C58  O14
  C70  C68  C71  O14
  C71  C64  C70  H35
  C71  C72  C68  H36
  H91  C68  C72  C69
  C72  C64  C69  H34
  C70  C69  C64  O11
  C49  C51  C52  O11
  C52  C53  C49  H27
  C49  C50  C53  O12
  C86  C85  C89  O12
  C89  C67  C86  H43
  C89  C88  C85  H45
  H90  C85  C88  C87
  C88  C67  C87  H44
  C86  C87  C67  O10
  C44  C45  C48  O10
  C48  C47  C44  H24
  C44  C46  C47   O9
  C79  C80  C83   O9
  C83  C82  C79  H42
  H84  C79  C82  C81
  C82  C66  C81  H41
  C81  C80  C66  O16
  C83  C66  C80  H40

DONE
STOP
```

nom.in

```
    0    0    2
mno.dat
MNO, OctaAcid, Methylated, neutral without carboxylate groups, RESP charges, UR
26/11-15
mno.dat
  MNO  INT     1
 CHANGE OMIT DU   BEG
   0.00000
   1  DUMM  DU    M     0.0000  0.0000   .0000   .0000
   2  DUMM  DU    M     0.0000  1.0000   .0000   .0000
   3  DUMM  DU    M     1.0000  1.0000   .0000   .0000
   4  C55   ca    M     4.846   1.737   1.522   -0.112539
   5  H28   ha    E     4.590   2.661   0.997    0.123080
   6  C42   ca    M     4.766   0.494   0.860   -0.112316
   7  C56   ca    S     5.121  -0.702   1.513   -0.112539
   8  H29   ha    E     5.072  -1.658   0.983    0.123080
   9  C9    c3    M     4.131   0.424  -0.526    0.122656
  10  O3    os    E     4.383   1.627  -1.253   -0.209810
  11  H19   h2    E     3.033   0.315  -0.400    0.132962
  12  O1    os    M     4.614  -0.705  -1.247   -0.209810
  13  C3    ca    M     3.635  -1.398  -1.964    0.027094
  14  C1    ca    M     3.150  -2.566  -1.376   -0.070050
  15  H1    ha    E     3.535  -2.887  -0.404    0.113670
  16  C4    ca    M     2.127  -3.288  -1.991    0.027094
  17  O2    os    E     1.656  -4.404  -1.295   -0.209810
  18  C5    ca    M     1.576  -2.882  -3.229    0.031393
  19  C7    c3    3     0.393  -3.684  -3.805   -0.005156
  20  H12   hc    E     0.391  -3.855  -4.892    0.066884
  21  C32   ca    S    -0.916  -3.124  -3.215    0.031393
  22  C31   ca    S    -1.694  -2.095  -3.796   -0.268186
  23  H56   ha    E    -1.371  -1.684  -4.760    0.163321
  24  H4    hc    E     0.490  -4.685  -3.346    0.066884
  25  C2    ca    M     2.115  -1.708  -3.809   -0.268186
  26  H2    ha    E     1.711  -1.370  -4.771    0.163321
  27  C6    ca    M     3.137  -0.937  -3.206    0.031393
  28  C8    c3    M     3.706   0.376  -3.778   -0.005156
  29  H13   hc    E     3.896   0.382  -4.862    0.066884
  30  H3    hc    E     4.699   0.472  -3.301    0.066884
  31  C10   ca    M     2.896   1.558  -3.213    0.031393
  32  C15   ca    S     1.731   2.099  -3.808   -0.268186
  33  H5    ha    E     1.403   1.695  -4.773    0.163321
  34  C14   ca    M     3.286   2.104  -1.967    0.027094
  35  C17   ca    M     2.561   3.135  -1.367   -0.070050
  36  H6    ha    E     2.874   3.524  -0.393    0.113670
  37  C13   ca    M     1.406   3.629  -1.973    0.027094
  38  O4    os    S     0.723   4.625  -1.270   -0.209810
  39  C34   c3    3    -0.424   4.173  -0.560    0.122656
  40  C41   ca    B    -0.515   4.855   0.803   -0.112316
  41  C50   ca    S    -1.778   5.013   1.408   -0.112539
  42  H25   ha    E    -2.690   4.776   0.853    0.123080
  43  C51   ca    S     0.670   5.179   1.494   -0.112539
  44  H26   ha    E     1.642   5.063   1.007    0.123080
  45  O5    os    E    -1.609   4.414  -1.309   -0.209810
  46  H18   h2    E    -0.327   3.079  -0.398    0.122656
  47  C16   ca    M     0.954   3.125  -3.217    0.031393
  48  C18   c3    M    -0.354   3.690  -3.806   -0.005156
  49  H14   hc    E    -0.352   3.868  -4.892    0.066884
  50  H7    hc    E    -0.450   4.689  -3.342    0.066884
  51  C19   ca    M    -1.538   2.886  -3.234    0.031393
  52  C23   ca    S    -2.078   1.710  -3.808   -0.268186
  53  H8    ha    E    -1.679   1.368  -4.770    0.163321
  54  C22   ca    M    -2.085   3.295  -1.997    0.027094
  55  C25   ca    M    -3.101   2.574  -1.370   -0.070050
  56  H9    ha    E    -3.482   2.900  -0.397    0.113670
  57  C21   ca    M    -3.585   1.403  -1.951    0.027094
  58  O6    os    S    -4.555   0.709  -1.225   -0.209810
  59  C38   c3    3    -4.059  -0.415  -0.503    0.122656
  60  C40   ca    B    -4.680  -0.464   0.894   -0.112316
  61  C45   ca    S    -5.069   0.741   1.514   -0.112539
  62  H22   ha    E    -5.047   1.681   0.956    0.123080
  63  C46   ca    S    -4.726  -1.690   1.589   -0.112539
```

64	H23	ha	E	-4.440	-2.621	1.089	0.123080
65	O7	os	E	-4.321	-1.622	-1.208	-0.209810
66	H20	h2	E	-2.959	-0.301	-0.398	0.132962
67	C24	ca	M	-3.094	0.939	-3.194	0.031393
68	C26	c3	M	-3.668	-0.376	-3.754	-0.005156
69	H15	hc	E	-3.869	-0.382	-4.835	0.066884
70	H10	hc	E	-4.658	-0.467	-3.269	0.066884
71	C28	ca	M	-2.854	-1.555	-3.191	0.031393
72	C30	ca	M	-3.234	-2.105	-1.944	0.027094
73	C33	ca	M	-2.515	-3.146	-1.358	-0.070050
74	H11	ha	E	-2.826	-3.546	-0.388	0.113670
75	C29	ca	M	-1.366	-3.638	-1.977	0.027094
76	O8	os	M	-0.675	-4.638	-1.289	-0.209810
77	C39	c3	M	0.458	-4.170	-0.562	0.122656
78	H21	h2	E	0.351	-3.075	-0.420	0.132962
79	C43	ca	M	0.523	-4.837	0.809	-0.112316
80	C61	ca	S	1.765	-4.935	1.467	-0.112539
81	H32	ha	E	2.688	-4.653	0.952	0.123080
82	C60	ca	M	-0.672	-5.216	1.454	-0.112539
83	H31	ha	E	-1.628	-5.153	0.926	0.123080
84	C63	ca	M	-0.614	-5.681	2.779	0.177924
85	O16	os	E	-1.700	-6.139	3.504	-0.281548
86	C59	ca	M	0.615	-5.768	3.448	-0.179464
87	H33	ha	E	0.649	-6.103	4.488	0.146982
88	C62	ca	M	1.802	-5.397	2.795	0.177924
89	O15	os	M	2.945	-5.578	3.539	-0.281548
90	C77	ca	M	4.186	-4.960	3.328	0.087409
91	C74	ca	S	4.322	-3.547	3.345	0.150694
92	C3M	c3	3	3.139	-2.617	3.462	-0.181608
93	H63	hc	E	2.277	-3.083	3.968	0.064532
94	H64	hc	E	2.776	-2.291	2.465	0.064532
95	H65	hc	E	3.374	-1.697	4.027	0.064532
96	C73	ca	M	5.288	-5.832	3.345	-0.145352
97	H39	ha	E	5.132	-6.916	3.382	0.158522
98	C76	ca	M	6.603	-5.337	3.389	-0.244274
99	H78	ha	E	7.462	-6.014	3.504	0.193636
100	C75	ca	M	6.771	-3.942	3.373	-0.145352
101	H38	ha	E	7.786	-3.533	3.432	0.158522
102	C65	ca	M	5.662	-3.078	3.355	0.087409
103	O13	os	M	5.960	-1.731	3.596	-0.281548
104	C57	ca	M	5.536	-0.650	2.856	0.177924
105	C54	ca	M	5.596	0.578	3.531	-0.179464
106	H30	ha	E	5.889	0.607	4.584	0.146982
107	C58	ca	M	5.260	1.766	2.865	0.177924
108	O14	os	M	5.438	2.909	3.620	-0.281548
109	C71	ca	M	4.853	4.160	3.414	0.087409
110	C70	ca	S	3.444	4.333	3.392	0.150694
111	C4M	c3	3	2.479	3.174	3.463	-0.181608
112	H66	hc	E	2.874	2.294	4.003	0.064532
113	H67	hc	E	2.196	2.823	2.451	0.064532
114	H68	hc	E	1.543	3.476	3.966	0.064532
115	C68	ca	M	5.743	5.248	3.476	-0.145352
116	H36	ha	E	6.822	5.070	3.545	0.158522
117	C72	ca	M	5.274	6.571	3.528	-0.244274
118	H91	ha	E	5.962	7.415	3.683	0.193636
119	C69	ca	M	3.884	6.772	3.469	-0.145352
120	H34	ha	E	3.497	7.795	3.533	0.158522
121	C64	ca	M	3.001	5.682	3.405	0.087409
122	O11	os	M	1.649	6.006	3.608	-0.281548
123	C52	ca	M	0.584	5.641	2.820	0.177924
124	C49	ca	M	-0.671	5.790	3.436	-0.179464
125	H27	ha	E	-0.730	6.133	4.473	0.146982
126	C53	ca	M	-1.845	5.480	2.732	0.177924
127	O12	os	M	-3.017	5.718	3.426	-0.281548
128	C89	ca	M	-4.243	5.073	3.223	0.087409
129	C86	ca	S	-4.358	3.660	3.298	0.150694
130	C1M	c3	3	-3.155	2.771	3.498	-0.181608
131	H57	hc	E	-2.380	3.282	4.098	0.064532
132	H58	hc	E	-2.683	2.491	2.538	0.064532
133	H59	hc	E	-3.432	1.830	4.007	0.064532
134	C85	ca	M	-5.366	5.916	3.187	-0.145352
135	H45	ha	E	-5.241	7.004	3.186	0.158522

136	C88	ca	M	-6.668	5.388	3.227	-0.244274
137	H90	ha	E	-7.544	6.048	3.312	0.193636
138	C87	ca	M	-6.809	3.989	3.252	-0.145352
139	H44	ha	E	-7.816	3.563	3.303	0.158522
140	C67	ca	M	-5.682	3.150	3.289	0.087409
141	O10	os	M	-5.942	1.803	3.570	-0.281548
142	C48	ca	M	-5.492	0.710	2.854	0.177924
143	C44	ca	M	-5.527	-0.502	3.560	-0.179464
144	H24	ha	E	-5.846	-0.515	4.607	0.146982
145	C47	ca	M	-5.152	-1.701	2.930	0.177924
146	O9	os	M	-5.281	-2.819	3.724	-0.281548
147	C83	ca	M	-4.791	-4.106	3.447	0.087409
148	C79	ca	M	-5.749	-5.133	3.439	-0.145352
149	H42	ha	E	-6.816	-4.898	3.513	0.158522
150	C82	ca	M	-5.356	-6.483	3.417	-0.244274
151	H84	ha	E	-6.094	-7.291	3.532	0.193636
152	C81	ca	M	-3.980	-6.763	3.341	-0.145352
153	H41	ha	E	-3.658	-7.810	3.335	0.158522
154	C66	ca	M	-3.027	-5.729	3.350	0.087409
155	C80	ca	M	-3.396	-4.361	3.438	0.150694
156	C2M	c3	3	-2.380	-3.265	3.640	-0.181608
157	H60	hc	E	-2.828	-2.402	4.161	0.064532
158	H61	hc	E	-1.977	-2.895	2.681	0.064532
159	H62	hc	E	-1.505	-3.596	4.224	0.064532

LOOP

C58	C55
C57	C56
C14	O3
C6	C3
C39	O2
C29	C32
C28	C31
C16	C15
C53	C50
C52	C51
C22	O5
C24	C23
C48	C45
C47	C46
C30	O7
C62	C61
C66	O16
C65	C74
C64	C70
C67	C86
C80	C83

IMPROPER

C58	C42	C55	H28
C9	C55	C42	C56
C57	C42	C56	H29
C1	C6	C3	O1
C3	C4	C1	H1
C1	C5	C4	O2
C7	C4	C5	C2
C7	C29	C32	C31
C28	C32	C31	H56
C5	C6	C2	H2
C8	C3	C6	C2
C8	C15	C10	C14
C10	C16	C15	H5
C10	C17	C14	O3
C14	C13	C17	H6
C17	C16	C13	O4
C34	C50	C41	C51
C53	C41	C50	H25
C52	C41	C51	H26
C18	C15	C16	C13
C18	C23	C19	C22
C19	C24	C23	H8

```
   C19  C25  C22   O5
   C22  C21  C25   H9
   C25  C24  C21   O6
   C38  C45  C40  C46
   C48  C40  C45  H22
   C47  C40  C46  H23
   C26  C23  C24  C21
   C26  C30  C28  C31
   C28  C33  C30   O7
   C30  C29  C33  H11
   C33  C32  C29   O8
   C39  C61  C43  C60
   C43  C62  C61  H32
   C43  C63  C60  H31
   C60  C59  C63  O16
   C63  C62  C59  H33
   C61  C59  C62  O15
   C74  C73  C77  O15
   C77  C65  C74  C3M
   C77  C76  C73  H39
   H78  C73  C76  C75
   C76  C65  C75  H38
   C74  C75  C65  O13
   C54  C56  C57  O13
   C57  C58  C54  H30
   C54  C55  C58  O14
   C70  C68  C71  O14
   C71  C64  C70  C4M
   C71  C72  C68  H36
   H91  C68  C72  C69
   C72  C64  C69  H34
   C70  C69  C64  O11
   C49  C51  C52  O11
   C52  C53  C49  H27
   C49  C50  C53  O12
   C86  C85  C89  O12
   C89  C67  C86  C1M
   C89  C88  C85  H45
   H90  C85  C88  C87
   C88  C67  C87  H44
   C86  C87  C67  O10
   C44  C45  C48  O10
   C48  C47  C44  H24
   C44  C46  C47   O9
   C79  C80  C83   O9
   C83  C82  C79  H42
   H84  C79  C82  C81
   C82  C66  C81  H41
   C81  C80  C66  O16
   C83  C66  C80  C2M

DONE
STOP
```

G1.in

```
    0    0    2

This is a remark line
molecule.res
G1   INT  0
CORRECT     OMIT DU   BEG
  0.0000
   1  DUMM  DU    M    0  -1  -2     0.000      .0        .0      .00000
   2  DUMM  DU    M    1   0  -1     1.449      .0        .0      .00000
   3  DUMM  DU    M    2   1   0     1.523   111.21       .0      .00000
   4  C1    c1    M    3   2   1     1.540   111.208  -180.000 -0.564113
   5  H1    ha    E    4   3   2     1.056    22.755    80.561  0.309304
   6  C2    c1    M    4   3   2     1.198   157.643   -99.090  0.021138
   7  C3    c3    M    6   4   3     1.434   179.242    66.974  0.036302
   8  H2    hc    E    7   6   4     1.125   109.097   -64.850  0.046452
   9  H3    hc    E    7   6   4     1.135   110.620    52.399  0.046452
  10  C4    c3    M    7   6   4     1.521   112.863   173.241  0.087275
  11  H4    hc    E   10   7   6     1.121   109.459   -56.280 -0.019439
  12  H5    hc    E   10   7   6     1.122   109.481    61.285 -0.019439
  13  C5    c3    M   10   7   6     1.511   111.014  -177.109 -0.081234
  14  H6    hc    E   13  10   7     1.120   109.068  -169.028 -0.044274
  15  H7    hc    E   13  10   7     1.119   109.964   -50.536 -0.044274
  16  C6    c     M   13  10   7     1.539   112.459    70.352  0.879953
  17  O2    o     E   16  13  10     1.264   117.846   114.011 -0.827051
  18  O1    o     M   16  13  10     1.267   118.511   -66.945 -0.827051


LOOP

IMPROPER
   C5   O2   C6   O1

DONE
STOP
```

G2.in

```
    0    0    2

This is a remark line
molecule.res
G2   INT  0
CORRECT     OMIT DU   BEG
  0.0000
   1  DUMM  DU    M    0  -1  -2     0.000      .0        .0      .00000
   2  DUMM  DU    M    1   0  -1     1.449      .0        .0      .00000
   3  DUMM  DU    M    2   1   0     1.523   111.21       .0      .00000
   4  C1    ca    M    3   2   1     1.540   111.208  -180.000 -0.179112
   5  H1    ha    E    4   3   2     1.102    58.501  -180.000  0.144645
   6  C2    ca    M    4   3   2     1.391   179.712   179.993 -0.160745
   7  H2    ha    E    6   4   3     1.099   120.376     0.007  0.144792
   8  C3    ca    M    6   4   3     1.403   119.821  -179.993 -0.036845
   9  C7    cg    S    8   6   4     1.419   120.109   180.000  0.375755
  10  N1    n1    E    9   8   6     1.165   180.000    90.000 -0.534385
  11  C4    ca    M    8   6   4     1.403   119.781     0.000 -0.160745
  12  H3    ha    E   11   8   6     1.099   119.803   180.000  0.144792
  13  C5    ca    M   11   8   6     1.391   119.821     0.000 -0.179112
  14  H4    ha    E   13  11   8     1.102   121.210   180.000  0.144645
  15  C6    ca    M   13  11   8     1.399   120.683     0.000  0.084991
  16  C8    c     M   15  13  11     1.525   120.395   180.000  0.756800
  17  O2    o     E   16  15  13     1.262   117.661   180.000 -0.772737
  18  O1    o     M   16  15  13     1.262   117.661    -0.000 -0.772737


LOOP
   C6   C1

IMPROPER
   C6   C2   C1   H1
   C1   C3   C2   H2
   C4   C2   C3   C7
   C5   C3   C4   H3
   C4   C6   C5   H4
   C8   C5   C6   C1
   C6   O2   C8   O1

DONE
STOP
```

G3.in

```
    0    0    2

This is a remark line
molecule.res
G3   INT  0
CORRECT     OMIT DU   BEG
  0.0000
   1  DUMM  DU    M    0  -1  -2     0.000      .0        .0      .00000
   2  DUMM  DU    M    1   0  -1     1.449      .0        .0      .00000
   3  DUMM  DU    M    2   1   0     1.523   111.21       .0      .00000
   4  C1    c3    M    3   2   1     1.540   111.208  -180.000 -0.350978
   5  H1    hc    E    4   3   2     1.116    90.975   -54.188  0.084387
   6  H2    hc    E    4   3   2     1.117    90.975    54.218  0.084387
   7  H13   hc    E    4   3   2     1.118   145.755  -180.000  0.084387
   8  C2    c3    M    4   3   2     1.507    35.684  -180.000  0.261609
   9  H3    hc    E    8   4   3     1.122   109.682  -121.277 -0.033521
  10  H4    hc    E    8   4   3     1.122   109.661   121.240 -0.033521
  11  C3    c3    M    8   4   3     1.515   111.224     0.000 -0.058263
  12  H5    hc    E   11   8   4     1.122   109.524    58.679  0.015459
  13  H6    hc    E   11   8   4     1.122   109.554   -58.732  0.015459
  14  C4    c3    M   11   8   4     1.515   110.983   180.000 -0.105818
  15  H7    hc    E   14  11   8     1.122   109.529    58.764  0.027822
  16  H8    hc    E   14  11   8     1.122   109.494   -58.829  0.027822
  17  C5    c3    M   14  11   8     1.518   110.574   180.000  0.266561
  18  H9    hc    E   17  14  11     1.122   109.221    58.904 -0.019084
  19  H10   hc    E   17  14  11     1.121   109.202   -58.943 -0.019084
  20  C6    c3    M   17  14  11     1.523   108.861   180.000 -0.289143
  21  H11   hx    E   20  17  14     1.128   109.253    59.347  0.152146
  22  H12   hx    E   20  17  14     1.129   109.239   -59.374  0.152146
  23  N1    n4    M   20  17  14     1.509   115.458   180.000  0.213768
  24  C8    c3    3   23  20  17     1.496   108.258   180.000 -0.452941
  25  H17   hx    E   24  23  20     1.122   109.358    60.041  0.209142
  26  H18   hx    E   24  23  20     1.123   109.396   180.000  0.209142
  27  H19   hx    E   24  23  20     1.122   109.386   -60.008  0.209142
  28  C9    c3    3   23  20  17     1.492   110.547    60.741 -0.452941
  29  H20   hx    E   28  23  20     1.122   109.316   174.717  0.209142
  30  H21   hx    E   28  23  20     1.123   109.545   -65.568  0.209142
  31  H22   hx    E   28  23  20     1.122   109.394    54.576  0.209142
  32  C7    c3    M   23  20  17     1.492   110.569   -60.689 -0.452941
  33  H14   hx    E   32  23  20     1.122   109.342   -54.598  0.209142
  34  H15   hx    E   32  23  20     1.122   109.563    65.505  0.209142
  35  H16   hx    E   32  23  20     1.123   109.342  -174.629  0.209142

LOOP

IMPROPER

DONE
STOP
```

G4.in

```
    0    0    2
g04.dat
G4 in SAMPL5 challenge, 2/12-15
g04.dat
  G4   INT     1
 CHANGE OMIT DU   BEG
   0.00000
   1  DUMM  DU    M     0.0000  0.0000   .0000   .0000
   2  DUMM  DU    M     0.0000  1.0000   .0000   .0000
   3  DUMM  DU    M     1.0000  1.0000   .0000   .0000
   4  C4    c3    M    22.150  18.674  22.919   0.062137
   5  H5    hc    E    21.352  18.242  23.527  -0.089187
   6  H6    hc    E    23.014  18.009  22.981  -0.089187
   7  C3    c3    M    22.438  20.095  23.471   0.021407
   8  H4    hc    E    22.698  19.957  24.523   0.050315
   9  C1    c3    M    21.249  21.039  23.369  -0.196293
  10  H1    hc    E    20.313  20.796  23.876   0.042411
  11  H2    hc    E    21.511  21.958  23.898   0.042411
  12  C2    c3    M    20.886  21.148  21.875   0.090325
  13  H3    hc    E    19.931  21.643  21.686   0.025680
  14  C5    c3    M    20.560  19.733  21.343  -0.549274
  15  H7    hc    E    20.026  19.676  20.393   0.101130
  16  H8    hc    E    19.925  19.338  22.139   0.101130
  17  C6    c3    M    21.765  18.780  21.431   1.616433
  18  C11   c     B    21.378  17.403  21.008  -0.855190
  19  O1    o     E    21.892  16.382  21.483  -0.322036
  20  O2    o     E    20.471  17.294  20.263  -0.322036
  21  C7    c3    M    22.846  19.485  20.603  -0.252302
  22  H9    hc    E    23.762  18.891  20.586  -0.039079
  23  H10   hc    E    22.595  19.549  19.542  -0.039079
  24  C10   c3    M    23.261  20.883  21.236   0.366020
  25  H14   hc    E    24.017  21.388  20.632  -0.009658
  26  C8    c3    B    22.117  21.907  21.386  -0.371892
  27  H11   hc    E    22.320  22.721  22.085   0.066101
  28  H12   hc    E    21.906  22.607  20.574   0.066101
  29  C9    c3    M    23.606  20.746  22.718  -0.575757
  30  H13   h1    E    24.010  21.713  23.029   0.227293
  31  BR1   br    E    25.133  19.607  23.199  -0.167925


LOOP
   C6   C4
   C9   C3
   C2   C8

IMPROPER
   C6   O1   C11  O2

DONE
STOP
```

G5.in

```
    0    0    2

This is a remark line
molecule.res
G5   INT  0
CORRECT     OMIT DU   BEG
  0.0000
   1  DUMM  DU    M    0  -1  -2     0.000      .0        .0      .00000
   2  DUMM  DU    M    1   0  -1     1.449      .0        .0      .00000
   3  DUMM  DU    M    2   1   0     1.523   111.21       .0      .00000
   4  C3    ca    M    3   2   1     1.540   111.208  -180.000 -0.249047
   5  H12   ha    E    4   3   2     1.101    68.865    -8.004  0.162324
   6  C2    ca    M    4   3   2     1.394   148.580   104.744 -0.111874
   7  H11   ha    E    6   4   3     1.101   119.891   -97.124  0.157142
   8  C1    ca    M    6   4   3     1.396   120.044    83.762 -0.124909
   9  H10   ha    E    8   6   4     1.101   119.989   179.407  0.155596
  10  C6    ca    M    8   6   4     1.394   119.911     0.107 -0.111874
  11  H14   ha    E   10   8   6     1.102   119.987   178.900  0.157142
  12  C5    ca    M   10   8   6     1.394   120.162    -0.188 -0.249047
  13  H13   ha    E   12  10   8     1.101   119.580   178.095  0.162324
  14  C4    ca    M   12  10   8     1.400   120.161    -0.172  0.214015
  15  C7    c3    M   14  12  10     1.486   120.296   177.309 -0.311862
  16  H15   hc    E   15  14  12     1.129   108.550  -126.339  0.150923
  17  H16   hc    E   15  14  12     1.123   110.713    -9.747  0.150923
  18  C8    c3    M   15  14  12     1.524   114.461   116.202 -0.184135
  19  H17   hx    E   18  15  14     1.129   109.257    51.432  0.168867
  20  H18   hx    E   18  15  14     1.130   107.999   169.737  0.168867
  21  N1    n4    M   18  15  14     1.511   117.005   -70.091  0.146884
  22  C10   c3    3   21  18  15     1.494   110.713    77.548 -0.440009
  23  H4    hx    E   22  21  18     1.125   109.327   -57.828  0.207530
  24  H5    hx    E   22  21  18     1.122   109.261  -178.010  0.207530
  25  H6    hx    E   22  21  18     1.122   109.374    61.990  0.207530
  26  C11   c3    3   21  18  15     1.490   110.986   -44.063 -0.440009
  27  H7    hx    E   26  21  18     1.123   109.396   -50.984  0.207530
  28  H8    hx    E   26  21  18     1.122   109.324  -171.009  0.207530
  29  H9    hx    E   26  21  18     1.124   109.419    69.326  0.207530
  30  C9    c3    M   21  18  15     1.497   108.213  -163.547 -0.440009
  31  H1    hx    E   30  21  18     1.122   109.522    65.160  0.207530
  32  H2    hx    E   30  21  18     1.123   109.329   -55.030  0.207530
  33  H3    hx    E   30  21  18     1.122   109.373  -174.888  0.207530


LOOP
   C4   C3

IMPROPER
   C4   C2   C3  H12
   C3   C1   C2  H11
   C2   C6   C1  H10
   C5   C1   C6  H14
   C4   C6   C5  H13
   C7   C5   C4   C3

DONE
STOP
```

G6.in

```
    0    0    2

This is a remark line
molecule.res
G6   INT  0
CORRECT     OMIT DU   BEG
  0.0000
   1  DUMM  DU    M    0  -1  -2     0.000      .0        .0      .00000
   2  DUMM  DU    M    1   0  -1     1.449      .0        .0      .00000
   3  DUMM  DU    M    2   1   0     1.523   111.21       .0      .00000
   4  O1    o     M    3   2   1     1.540   111.208  -180.000 -0.485836
   5  N1    no    M    4   3   2     1.204    89.524  -180.000  0.763774
   6  O2    o     E    5   4   3     1.208   120.634  -179.945 -0.485836
   7  C1    ca    M    5   4   3     1.479   120.005     0.000  0.048672
   8  C2    ca    M    7   5   4     1.405   120.109    -0.000 -0.197997
   9  H1    ha    E    8   7   5     1.105   121.969    -0.000  0.183442
  10  C4    ca    M    8   7   5     1.397   119.237   180.000  0.042852
  11  C7    c     B   10   8   7     1.529   120.217   180.000  0.759149
  12  O3    o     E   11  10   8     1.261   117.564    -0.000 -0.771486
  13  O4    o     E   11  10   8     1.261   117.167   180.000 -0.771486
  14  C6    ca    M   10   8   7     1.396   119.868     0.000 -0.150203
  15  H4    ha    E   14  10   8     1.103   118.123   180.000  0.147987
  16  C5    ca    M   14  10   8     1.398   120.583     0.000 -0.108897
  17  H3    ha    E   16  14  10     1.099   120.050   180.000  0.119989
  18  C3    ca    M   16  14  10     1.390   120.415     0.000 -0.256571
  19  H2    ha    E   18  16  14     1.101   120.733   180.000  0.162449


LOOP
   C3   C1

IMPROPER
   C1   O1   N1   O2
   C3   C2   C1   N1
   C1   C4   C2   H1
   C7   C6   C4   C2
   C4   O3   C7   O4
   C5   C4   C6   H4
   C6   C3   C5   H3
   C5   C1   C3   H2

DONE
STOP
```

G2.dat

```
Parameters for Sample5 ligands, 25/11-15

DIHE
ca-ca-cg-n1   1      0.000      0.000    2.   !    0.00 2 1
```

Figure S1. Geometric measures employed to analyse the structures,.

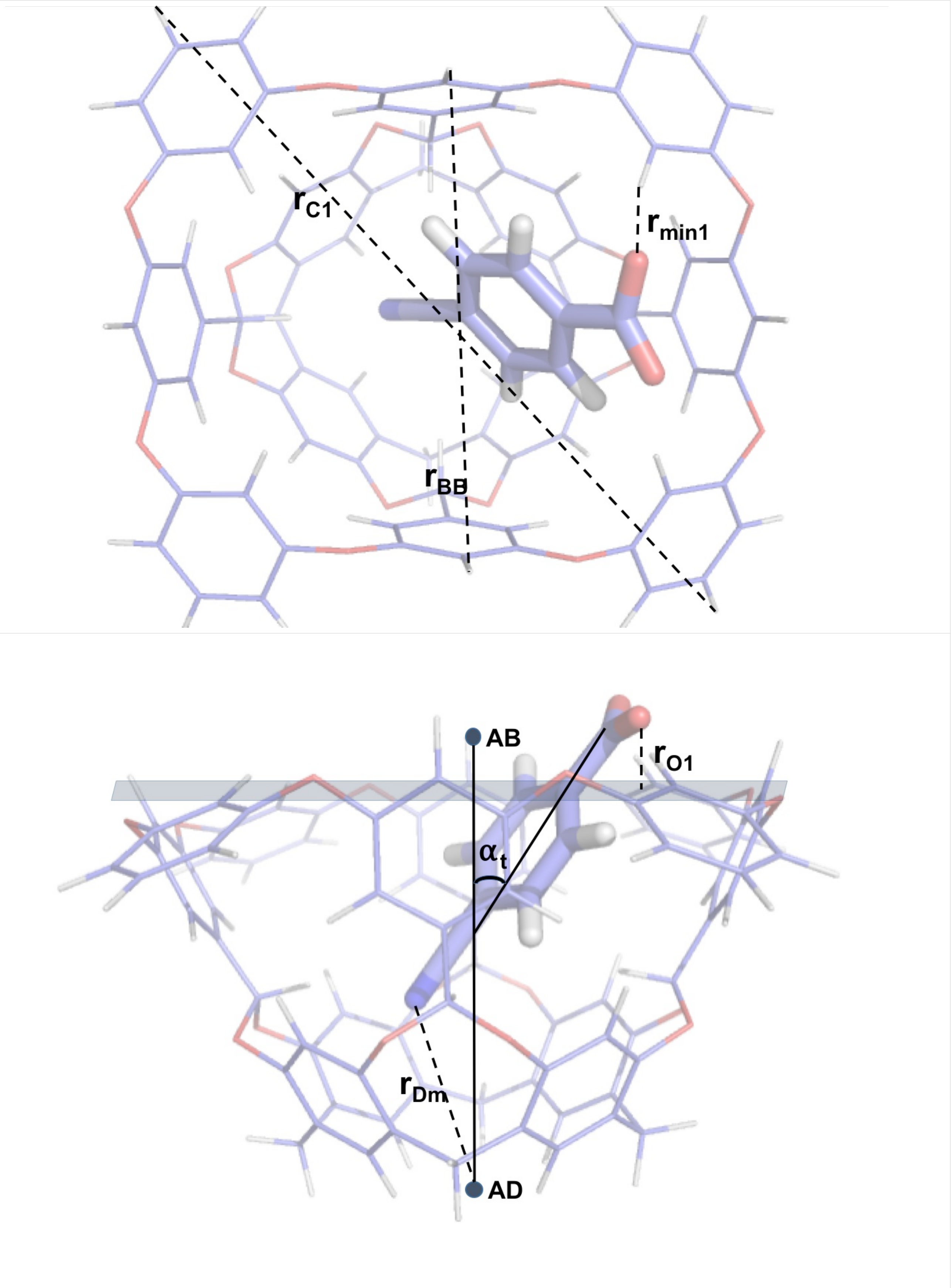

Figure S2. Typical snapshot of G1 in NOH during the MD simulation.

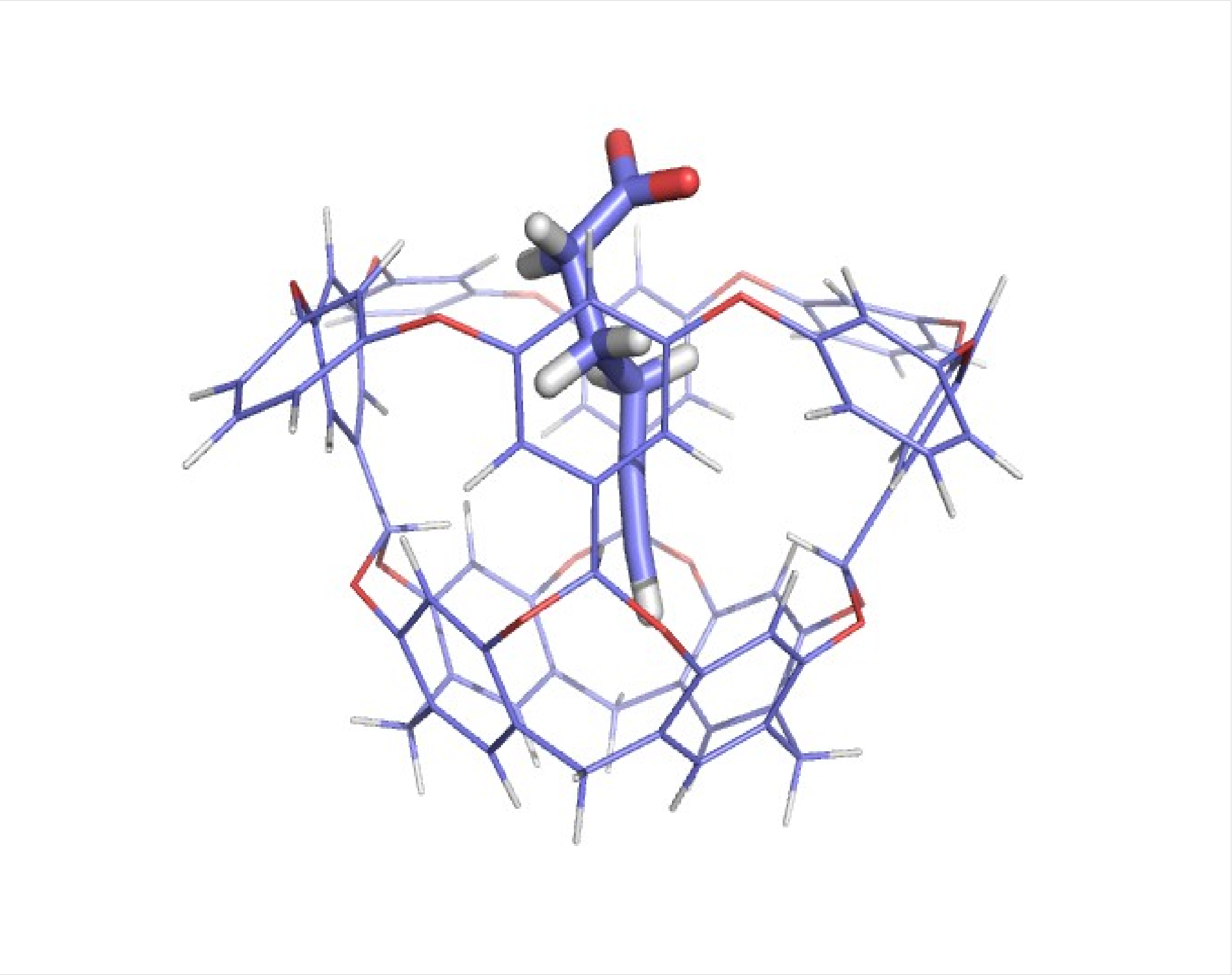

Supplement: Supplementary file 1 — Supplementary material 1 (PDF 448 kb) [file 10822_2016_9957_MOESM1_ESM.pdf]
